# Cytotoxicity Evaluation of Chalcones and Flavanones from the Leaves of *Corema album* (L.) D. Don

**DOI:** 10.3390/biom16040509

**Published:** 2026-03-28

**Authors:** Antonio Canoyra, Nuria Acero, Dolores Muñoz-Mingarro, Antonio J. León-González, José Luis Espartero, Carmen Martín-Cordero

**Affiliations:** 1 Pharmaceutical and Health Science Department, Pharmacy Faculty, San Pablo-CEU University, CEU Universities, Urbanización Montepríncipe, Boadilla del Monte, 28660 Madrid, Spain; a.canoyra@usp.ceu.es (A.C.); nacemes@ceu.es (N.A.); 2 Chemistry and Biochemistry Department, Pharmacy Faculty, San Pablo-CEU University, CEU Universities, Urbanización Montepríncipe, Boadilla del Monte, 28660 Madrid, Spain; dmumin@ceu.es; 3 Department of Pharmacology, Faculty of Pharmacy, University of Seville, C/P. García González, 2, 41012 Seville, Spain; ajleon@us.es; 4 Department of Organic and Pharmaceutical Chemistry, Faculty of Pharmacy, University of Seville, 41012 Sevilla, Spain; jles@us.es

**Keywords:** *Corema album*, chalcones, flavanones, 2′,4′-dihydroxychalcone, pinocembrin, cytotoxicity

## Abstract

In a preliminary screening of phytochemical compounds from Andalusian vascular plants, the ethyl acetate extract obtained from the leaves of *Corema album* (L.) D. Don (Ericaceae) was selected due to its cytotoxic activity. Eight phenolic compounds were isolated from this extract: four chalcones (2′,4′-dihydroxydihydrochalcone, 2′,4′-dihydroxychalcone, 2′,4′-dihydroxy-6′-methoxydihydrochalcone, 2′-methoxy-4′-hydroxydihydrochalcone) and four flavanones (pinocembrin, 6-methylpinocembrin, 6,8-dimethylpinocembrin and 7-O-prenylpinocembrin). Their structures were elucidated using ^1^H NMR and ^13^C NMR data, including 2D NMR, as well as mass spectrometry. These compounds were evaluated using the MTT cytotoxicity assay against human colorectal adenocarcinoma (HT-29) and renal adenocarcinoma (ACHN) cell lines. The chalcone 2′,4′-dihydroxychalcone exhibited greater cytotoxicity than the corresponding 2′,4′-dihydroxydihydrochalcone in both cell lines. The IC_50_ values (µM ± SEM) were 13.31 ± 0.48 and 9.43 ± 0.34 for the chalcone, and 62.23 ± 1.06 and 54.68 ± 1.62 for the dihydrochalcone, respectively. The introduction of methyl groups at positions 6 and 8 of pinocembrin increased cytotoxicity in the ACHN cell line. The IC_50_ values (µM ± SEM) were 91.28 ± 3.03 for pinocembrin, 64.36 ± 0.53 for 6-methylpinocembrin, and 28.74 ± 0.35 for 6,8-dimethylpinocembrin. These results highlight the leaves of *C. album* as a promising source of chalcones and flavanones with pharmacological interest.

## 1. Introduction

*Corema album* (L.) D. Don (Ericaceae) is a dioecious, densely branched shrub with erect stems reaching up to 1 m in height. Its fruits are edible berries with a slightly acidic flavor, and they have traditionally been used as refreshing agents as well as for their vermifuge and febrifugal properties. The species grows on sandy soils and coastal dunes along the Atlantic coast of the Iberian Peninsula, from La Coruña to Cádiz [1].

**Figure 1:**
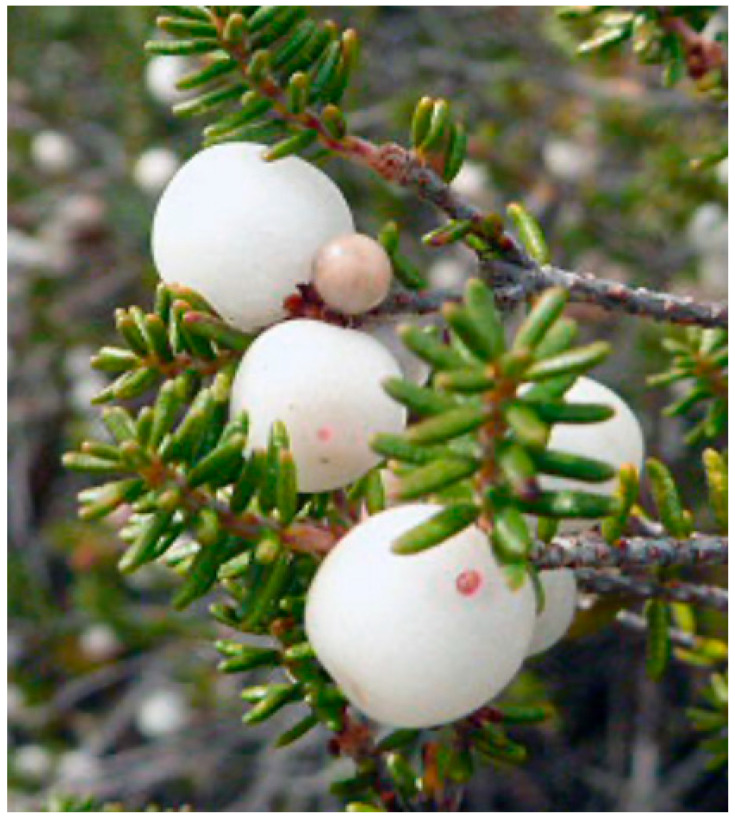
*Corema album* (L.) D. Don.

*Corema album* fruits (Figure 1) have attracted attention due to their potential health benefits. Recent studies have shown that juice from its berries enhances the viability of dopaminergic and cholinergic cells exposed to neurotoxic agents and exhibits antioxidant properties in neuronal cells [2]. In addition, twelve phenolic compounds have been identified in berry juice, including four phenolic acids (5-O-caffeoylquinic acid, caffeic acid, phloretic acid, ellagic acid), two flavanones (naringin, pinocembrin), three flavonols (myricetin-3-O-galactoside, quercetin-3-O-galactoside, kaempferol-3-O-glucoside), two flavanols ((-)-epigallocatechin, (-)-epicatechin), and one coumarin (6,7-dihydroxycoumarin) [2]. Additionally, due to its rich composition of the essential fatty acid α-linolenic acid (omega-3), the low proportion of n-6/n-3 fatty acids, and the low values of IA (index of atherogenicity) and IT (index of thrombogenicity), *C. album* seed oil also exhibits potential health benefits [3].

Chalcones are naturally occurring precursors of flavonoid biosynthesis that exhibit anticancer activity through multiple mechanisms, making them promising candidates for cancer treatment and prevention [4]. As α, β-unsaturated ketones, chalcones act as Michael acceptors, preferentially reacting with thiol groups such as cysteine residues in proteins. These interactions enable modulation of signaling pathways involved in carcinogenesis and contribute to a lower toxicity compared with other anticancer agents due to reduced reactivity with amino and hydroxyl groups [5].

Flavanones, a subclass of flavonoids, are recognized for their potent anticancer, antioxidant, and anti-inflammatory properties [6]. Pinocembrin (PB) is a flavanone with promising anticancer potential. It modulates several key signaling pathways, including PI3K/AKT, MAPK, and mTOR, leading to apoptosis, cell-cycle arrest, and inhibition of angiogenesis and metastasis. Preclinical studies have shown dose-dependent cytotoxicity in several cancer cell lines, along with significant tumor growth suppression in xenograft and syngeneic models. Toxicity studies in rodents report no acute or sub-acute effects, and early human trials suggest good tolerability. Overall, PB demonstrates strong anticancer potential and a favorable safety profile, supporting further clinical investigation [7].

As part of a preliminary screening program for antitumoral compounds isolated from Andalusian vascular plants, the ethyl acetate extract from *C. album* leaves was shown to be active against HT-29 cells. We isolated two cytotoxic dihydrochalcones: 2′,4′-dihydroxydihydrochalcone and 2′-methoxy-4′-hydroxydihydrochalcone [8].

The aim of this study was to isolate and identify active compounds from the ethyl acetate extract of *C. album* leaves and to determine their cytotoxic activity against HT-29 (human colorectal adenocarcinoma) and ACHN (renal adenocarcinoma) cell lines. In conclusion, 2′,4′-dihydroxychalcone showed greater cytotoxicity than its dihydrochalcone counterpart and natural derivatives in the two cell lines tested, likely due to the α, β-unsaturation that increases its electrophilicity and reactivity. These findings support the role of the α, β-unsaturated system in enhancing the anticancer potential of chalcone derivatives. In vitro cytotoxicity assays also showed that natural pinocembrin exhibits moderate antiproliferative activity in the tested tumor cell lines, with IC_50_ values above 100 µM. However, methylated derivatives of pinocembrin achieved significantly lower IC_50_ values (around 28–60 µM) in resistant cell lines such as ACHN, indicating that structural modification enhances the cytotoxic potential of this natural flavanone. These results are particularly promising, given that the ACHN cell line is widely used as a model of renal cancer to investigate mechanisms of chemoresistance and to evaluate novel therapeutic agents.

## 2. Materials and Methods

### 2.1. Plant Material

Leaves of wild *C. album* were collected in May 2024 in Huelva, Spain (37°04′10.15″ N, 6°41′15.45″ W). Botanical identification was performed by Dr. Mari Cruz Díaz-Barradas from the Department of Plant Biology and Ecology at the University of Seville. A voucher specimen (no. 277798) was deposited in the University of Seville Herbarium as a reference. The plant material was subsequently air-dried at room temperature.

### 2.2. Chemicals

In this study, the following solvents were used: ethyl acetate for extraction; hexane and ethyl acetate in various mixtures as eluting systems for the separation procedure on silica gel 60 (Art. No. 7734, Merck, Darmstadt, Germany) columns and as mobile phases for TLC; UV light (254 and 366 nm), AlCl_3_·6H_2_O, and oleum for detection in TLC; DMSO-*d*_6_ as solvent for the identification of metabolites by NMR spectroscopy; MTT (3-(4,5-dimethylthiazol-2-yl)-2,5-diphenyltetrazolium bromide) for the cytotoxicity assay; and DMSO as solvent for dissolution of test compounds. All solvents were of RPE grade unless otherwise specified and were purchased from PanReac AppliChem (Barcelona, Spain). Deuterated solvents, DMSO, silica gel and reagents for cytotoxicity assays were purchased from Merck (Darmstadt, Germany).

### 2.3. Extraction

The dried leaves (250 g) of female plants of *C. album* were extracted in an ultrasonic bath for 45 min at 40 °C using ethyl acetate. After filtration, the solvent was removed under reduced pressure using a rotary evaporator at 45 °C to afford the crude ethyl acetate extract (4.8% *w*/*w* yield).

### 2.4. Isolation and Identification of the Compounds

The crude extract (5 g) was subjected to silica gel column chromatography (5 × 50 cm) and eluted with gradient mixtures of n-hexane/ethyl acetate from 99:1 to 50:50 (*v*/*v*). A total of 950 fractions (10 mL each) were collected, allowing the isolation of eight compounds. All compounds were identified by comparison of their spectroscopic data with those reported in the literature [9,10,11,12,13,14,15,16,17] and with authentic standards available in our laboratory. All NMR (^1^H, ^13^C, COSY, HSQC, HMBC) and MS spectra used for the structural identification of the isolated compounds are provided in the Supplementary Materials (Figures S1–S35). In particular, the identified compounds, in order of elution from the column, were as follows: 7-O-prenyl-pinocembrin (**1**), isolated by crystallization as white needles from fractions 41–100 (20 mg); 6,8-dimethylpinocembrin (**2**), as white needles from fractions 135–200 (8 mg); 6-methylpinocembrin (**3**) as white needles from fractions 230–240 (7 mg); 2′,4′-dihydroxydihydrochalcone (**4**) as white needles from fractions 260–319 (34 mg); pinocembrin (**5**) as pale yellow needles from fractions 371–590 (25 mg); 2′,4′-dihydroxychalcone (**6**) as yellow needles from fractions 600–650 (59 mg); 2′,4′-dihydroxy-6′-methoxydihydrochalcone (**7**) as white needles from fractions 670–790 (10 mg); and 2′-methoxy-4′-hydroxydihydrochalcone (**8**) as white needles from fractions 800–820 (45 mg).

### 2.5. NMR Analysis

NMR spectra were recorded on a Bruker Avance III spectrometer (Bruker BioSpin AG, Fällanden, Switzerland), equipped with a TCI HCN Z-gradient cryoprobe, operating at 500.13 MHz for ^1^H and 125.75 MHz for ^13^C. Chemical shifts (δ) were reported in parts per million (ppm) and referenced to the residual solvent signals (δ 2.50 for ^1^H and δ 39.52 for ^13^C). Samples were dissolved in dimethyl sulfoxide-*d*_6_ (DMSO-*d*_6_), and spectra were recorded at 303 K. Two-dimensional NMR experiments (COSY, NOESY, HSQC, and HMBC) were performed to achieve complete signal assignment in the ^1^H and ^13^C NMR spectra.

### 2.6. MS Analysis

MS spectra were acquired on a high-resolution magnetic sector mass spectrometer Q Exactive GC (Thermo Scientific, Bremen, Germany) equipped with an electron ionization (EI) source operating at 70 eV. Samples were introduced using a direct insertion probe (DIP). The ion source and probe temperatures were both set to 250 °C to ensure adequate volatilization of the samples. Spectra were recorded in full-scan mode over an *m*/*z* range of 50–800. Mass calibration was performed using perfluorotributylamine (PFTBA), and data acquisition and processing were carried out with Xcalibur Qual Browser software version 4.1 (Thermo Scientific, Bremen, Germany).

### 2.7. NMR and MS Data of the Isolated Compounds

7-O-prenyl-pinocembrin (**1**): was obtained as colorless needles. ^1^H NMR (500 MHz, DMSO-*d*_6_): δ 12.08 (s, 1H, OH-5), 7.52 (m, 2H, H-2′, H-6′), 7.44 (m, 3H, H-3′, H-4′, H-5′), 6.13 (d, *J* = 2.3 Hz, 1H, H-8), 6.08 (d, *J* = 2.3 Hz, 1H, H-6), 5.61 (dd, *J* = 12.7, 3.1 Hz, 1H, H-2), 5.39 (tq, *J* = 6.8, 1.3 Hz, 1H, H-2″), 4.57 (d, *J* = 6.8 Hz, 2H, H-1a″, H-1b″), 3.29 (dd, *J* = 17.1, 12.7 Hz, 1H, H-3a), 2.81 (dd, *J* = 17.1, 3.1 Hz, 1H, H-3b), 1.73 (d, *J* = 1.1 Hz, 3H, Me-5″), 1.68 (d, *J* = 1.3 Hz, 3H, Me-4″). ^13^C NMR (125 MHz, DMSO-*d*_6_) δ 196.4 (C-4), 166.7 (C-7), 163.1 (C-5), 162.6 (C-9), 138.6 (C-1′), 138.1 (C-3″), 128.5 (C-3′, C-4′, C-5′), 126.6 (C-2′, C-6′), 119.0 (C-2″), 102.5 (C-10), 95.3 (C-6), 94.4 (C-8), 78.5 (C-2), 65.1 (C-1″), 42.1 (C-3), 25.3 (Me-5″), 18.0 (Me-4″). MS (EI) *m*/*z* (rel. int., %): 324 (M^+^•, 22), 309 (10), 256 (38), 255 (99), 238 (20), 179 (100), 152 (33). To date, this O-prenylated flavanone has been reported exclusively from species of *Helichrysum* and from *Metalasia cymbifolia* (Asteraceae) [9,10].

6,8-dimethylpinocembrin (**2**): colorless needles. ^1^H NMR (500 MHz, DMSO-*d_6_*): δ 12.36 (s, 1H, OH-5), 9.78 (br s, 1H, OH-7), 7.52 (m, 2H, H-2′, H-6′), 7.42 (m, 2H, H-3′, H-5′), 7.36 (m, 1H, H-4′), 5.54 (dd, *J* = 12.3, 3.2 Hz, 1H, H-2), 3.16 (dd, *J* = 17.0, 12.3 Hz, 1H, H-3a), 2.84 (dd, *J* = 17.0, 3.2 Hz, 1H, H-3b), 1.96 (s, 3H, Me-6), 1.94 (s, 3H, Me-8); ^13^C NMR (125 MHz, DMSO-*d*_6_): δ 196.7 (C-4), 163.4 (C-7), 158.9 (C-5), 157.6 (C-9), 139.7 (C-1′), 129.0 (C-3′, C-5′), 128.8 (C-4′), 126.7 (C-2′, C-6′), 103.8 (C-10), 103.1 (C-8), 102.1 (C-6), 78.3 (C-2), 42.6 (C-3), 8.8 (Me-6), 8.1 (Me-8). MS (EI) *m*/*z* (rel. int., %): 152 (100), 284 (M^+^•, 90), 207 (52), 180 (46), 165 (43), 269 (41), 205 (38), 266 (7). This doubly C-methylated flavanone (5,7-dihydroxy-6,8-dimethylflavanone or demethoxymatteucinol) has been previously isolated from *Empetrum nigrum*, *Ceratiola ericoides, Matteuccia* spp. and *Syzygium* spp. [11,15,18,19].

6-methylpinocembrin (**3**): colorless needles. ^1^H NMR (500 MHz, DMSO-*d*_6_) δ 12.39 (s, 1H, OH-5), 10.88 (br s, 1H, OH-7), 7.50 (m, 2H, H-2′, H-6′), 7.39 (m, 3H, H-3′, H4′, H-5′), 5.99 (s, 1H, H-8), 5.53 (dd, *J* = 12.5, 3.2 Hz, 1H, H-2), 3.21 (dd, *J* = 17.1, 12.5 Hz, 1H, H-3a), 2.77 (dd, *J* = 17.1, 3.2 Hz, 1H, H-3b), 1.87 (s, 3H, Me-6); ^13^C NMR (125 MHz, DMSO-*d*_6_*)*: δ 195.7 (C-4), 165.0 (C-7), 160.7 (C-5), 160.1 (C-9), 138.8 (C-1′), 128.5 (C-3′, C-5′), 128.4 (C-4′), 126.5 (C-2′, C-6′), 103.3 (C-6), 101.3 (C-10), 94.3 (C-8), 78.2 (C-2), 42.2 (C-3), 6.9 (Me-6); MS (EI) m/z (rel. int., %): 138 (100), 193 (92), 269 (M^+^•, 54), 270 (35), 166 (33), 110 (31). This compound is also known as strobopinin (5,7-dihydroxy-6-methylflavanone), originally isolated from the heartwood of *Pinus strobus* [20].

2′,4′-dihydroxydihydrochalcone (**4**): colorless needles. ^1^H NMR (500 MHz, DMSO-*d*_6_): δ 12.56 (s, 1H, OH-2′), 10.61 (br s, 1H, OH-4′), 7.80 (d, *J* = 8.9 Hz, 1H, H-6′), 7.25 (m, 5H, H-2″–H-6″), 6.34 (dd, *J* = 2.3, 8.9 Hz, 1H, H-5′), 6.23 (d, *J* = 2.3 Hz, 1H, H-3′), 3.28 (t, *J* = 7.6 Hz, 2H, H-2), 2.92 (t, *J* = 7.6 Hz, 2H, H-3). ^13^C NMR (125 MHz, DMSO-d_6_) δ 203.5 (C-1), 164.7 (C-4′), 164.1 (C-2′), 141.0 (C-1″), 133.0 (C-6′), 128.3 (C-3″, C-5″), 128.2 (C-2″, C-6″), 125.9 (C-4″), 112.5 (C-1′), 108.1 (C-5′), 102.3 (C-3′), 38.9 (C-2), 29.7 (C-3). MS (EI) *m/z* (rel. int., %): 137 (100), 242 (M^+^•, 18), 224 (15), 110 (10), 138 (8), 81 (7), 147 (6), 91 (6). This compound has been previously isolated and identified from the leaves of *Empetrum nigrum* [11].

Pinocembrin (**5**): colorless needles. ^1^H NMR (500 MHz, DMSO-*d*6): ^1^H NMR (500 MHz, DMSO d_6_): δ 12.11 (br s, 1H, OH-5), 10.82 (s, 1H, OH-7), 7.36–7.51 (m, *J* = 7.5 Hz, 5H, H-2′, H-3′, H-4′, H-5′, H-6′), 5.91 (d, J = 2.0 Hz, 1H, H-8), 5.88 (d, *J* = 2.1 Hz, 1H, H-6), 5.58 (dd, *J* = 12.6, 3.1 Hz, 1H, H-2), 3.25 (dd, *J* = 17.1, 12.6 Hz, 1H, H-3a), 2.77 (dd, *J* = 17.1, 3.1 Hz, 1H, H-3b). ^13^C NMR (125 MHz, DMSO-*d*_6_) δ 195.9 (C-4), 166.7 (C-7), 163.4 (C-9), 162.7 (C-5), 138.7 (C-1′), 128.5 (C-3′, C-4′, C-5′), 126.6 (C-2′, C-6′), 101.7 (C-10), 95.9 (C-6), 95.0 (C-8), 78.3 (C-2), 42.0 (C-3). MS (EI) *m*/*z* (rel. int. %): 179 (100), 255 (97), 256 (M^+^•, 58), 152 (44), 124 (44), 238 (20), 137 (7), 103 (7), 96 (10), 77 (5).

2′,4′-dihydroxychalcone (**6**): Yellow needles. The identity of this product is confirmed by its ^1^H NMR (500 MHz, DMSO-d_6_) δ 13.39 (s, 1H, OH-2′), 8.19 (d, *J* = 9.0 Hz, 1H, H-6′), 7.96 (d, *J* = 15.4 Hz, 1H, H-3), 7.89 (m, 2H, H-2” H-6”), 7.78 (d, *J* = 15.4 Hz, 1H, H-2), 7.46 (m, 3H, H3″H4″H5″), 6.41 (dd, *J* = 9.0, 2.4 Hz, 1H, H-5′), 6.29 (d, J = 2.4 Hz, 1H, H-3′). ^13^C NMR (125 MHz, DMSO-*d*_6_) δ 191.4 (C-1), 165.8 (C-4′), 165.4 (C-2′), 143.6 (C-3), 134.6 (C-1″), 133.1 (C-6′), 130.6 (C-4″), 129.0 (C-3″, C-5″), 128.9 (C-2″, C-6″), 121.3 (C-2), 113.0 (C-1′), 108.3 (C-5′), 102.6 (C-3′). MS (EI) *m*/*z* (rel. int. %): 240 (M^+^•, 25), 239 (100), 163 (88), 240 (25), 137 (25), 223 (10). This compound has been previously isolated from phylogenetically closely related species such as *Empetrum nigrum* and *Ceratiola ericoides*, as well as from more distantly related plant taxa [11,15,21].

2′,4′-Dihydroxy-6′-methoxy-dihydrochalcone (**7**): Colourless needles. ^1^H NMR (500 MHz, DMSO-*d*_6_): δ 13.7 (s, 1H, OH-2′), 10.7 (br s, 1H, OH-4′), 7.27 (m, 5H, H-2″-H-6″), 5.94 (d, *J* = 1.8 Hz, 1H, H-5′), 5.83 (d, *J* = 1.8 Hz, 1H, H-3′), 3.79 (s, 3H, OMe-2′), 3.21 (t, *J* = 7.7 Hz, 2H, H-2), 2.86 (t, *J* = 7.7 Hz, 2H, H-3); ^13^C-NMR (125 MHz, DMSO-*d*_6_): δ 203.2 (C=O), 166.1 (C-2′), 165.4 (C-4′), 163.0 (C-6′), 141.5 (C-1″), 125.8 (C-4″), 128.3 (C-2″, C-3″, C-5″, C-6″),104.1 (C-1′), 95.8 (C-3′), 91.6 (C-5′), 55.8 (OCH_3_), 45.0 (C-2), 30.2 (C-3). MS (EI) *m*/*z* (rel. int. %): 167 (100), 140 (19), 272 (M^+^•, 18), 255 (11), 241 (8), 137 (7), 121 (7), 152 (6), 181 (4), 91 (4). It has previously been isolated from species of *Empetrum* and *Ceratiola* (Ericaceae) [11,15] and derivatives have also been reported in more distantly related genera such as *Lotus* (Fabaceae). It has been named uvangoletin because it was isolated for the first time from *Uvaria acuminata* [22].

2′-methoxy-4′-hydroxydihydrochalcone (**8**): Colorless needles. ^1^H NMR (500 MHz, DMSO-*d*_6_): δ 10.25 (s, 1H, OH-4′), 7.56 (d, *J* = 8.5 Hz, 1H, H-6′), 7.25 (t, *J* = 7.4 Hz, 2H, H-3″, H-5″), 7.21 (d, *J* = 7.4 Hz, 2H, H-2″, H-6″), 7.15 (tt, *J* = 7.4, 1.4 Hz, 1H, H-4″), 6.46 (d, *J* = 2.2 Hz, 1H, H-3′), 6.41 (dd, *J* = 8.5, 2.2 Hz, 1H, H-5′), 3.81 (s, 3H, OMe-2′), 3.14 (t, *J* = 7.6 Hz, 2H, H-2), 2.85 (t, *J* = 7.6 Hz, 2H, H-3); ^13^C-NMR (125 MHz, DMSO-*d*_6_): δ 197.7 (C=O), 163.0 (C-4′), 160.9 (C-2′), 141.7 (C-1″), 132.0 (C-6′), 128.2 (C-2″, C-3″, C-5″, C-6″), 125.6 (C-4″), 118.2 (C-1′), 107.8 (C-5′), 99.0 (C-3′), 55.5 (OCH_3_), 44.6 (C-2), 30.0 (C-3). MS *m*/*z* (reI. int. %): 151 (100), 124 (10), 256 (M^+^•, 9), 137 (4), 108 (4), 255 (2), 91 (2). This compound has been previously isolated from leaves of *E. nigrum* [11].

### 2.8. Cell Lines

The antiproliferative properties of the isolated chalcones and flavanones were evaluated in human colorectal (HT-29) and renal (ACHN) cancer cell lines. All cell lines were obtained from the European Collection of Authenticated Cell Cultures (ECACC, Salisbury, UK). HT-29 cells were maintained in McCoy’s 5A medium supplemented with 10% fetal bovine serum (FBS), 2 mM L-glutamine, and 1% penicillin–streptomycin (100 U/mL penicillin and 100 µg/mL streptomycin). ACHN cells were grown in minimum essential medium (MEM) enriched with 10% FBS, 2 mM L-glutamine, 1% non-essential amino acids (NEAA), and 1% penicillin–streptomycin (100 U/mL penicillin and 100 µg/mL streptomycin). All cultures were kept at 37 °C in a humidified incubator with 5% CO_2_.

### 2.9. MTT Colorimetric Assay

The cytotoxic capacity of the compounds isolated from *C. album* leaves was studied using the MTT assay (3-(4,5-dimethylthiazol-2-yl)-2,5-diphenyltetrazolium bromide). This colorimetric assay evaluates mitochondrial enzyme activity through reduction of the yellow MTT dye to violet formazan crystals. According to the protocol, exponentially growing cells were harvested and seeded (1 × 10^4^ cells/well) in 96-well plates (100 µL/well). After 24 h of incubation, cells were treated with serial dilutions of the compounds (100–1 µM). After 72 h of exposure, 10 µL of filter-sterilized MTT solution (5 mg/mL in PBS) was added to each well. Following a further 4 h incubation, the resulting formazan crystals were dissolved in DMSO, and absorbance was measured at 570 nm. Untreated cells (100% viability) were used as the negative control, and doxorubicin was used as the positive control. IC_50_ values were determined from dose–response curves generated from the viability data. All experiments were performed in three independent biological replicates, and each treatment condition was evaluated with eight technical replicates per experiment to ensure reproducibility.

### 2.10. Statistical Methods

Collected absorbance values were corrected for background absorbance using wells containing medium, MTT, and DMSO (no cells) and normalized to the mean value of the untreated control (100% viability). Data were fitted to concentration–response curves and analyzed using nonlinear regression in GraphPad Prism 8.0 (GraphPad Software, San Diego, CA, USA).

## 3. Results

### 3.1. Phytochemistry

**Figure 2:**
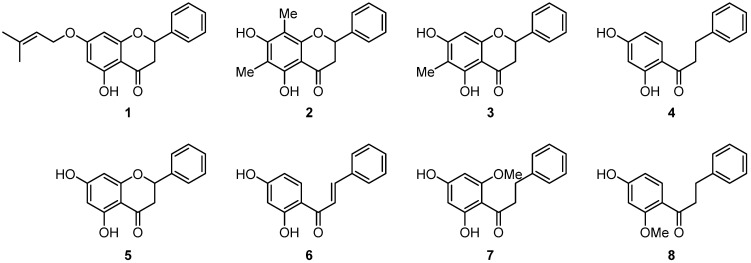
Chemical structures of the isolated flavanones, chalcone and dihydrochalcones from the leaves of *C. album*.

The phytochemical investigation of the cytotoxic ethyl acetate extract obtained from the leaves of *C. album* resulted in the isolation and identification of eight phenolic compounds, including flavanones, a chalcone, and dihydrochalcones. Structural elucidation was performed by NMR and EI-MS analyses and confirmed by comparison with literature data and authentic standards. The isolated compounds were listed according to increasing polarity, consistent with their elution order. Among the flavanones, pinocembrin (**5**) was the predominant constituent, together with three less polar derivatives: one prenylated analogue, 7-O-prenyl-pinocembrin (**1**), and two C-methylated derivatives, 6,8-dimethylpinocembrin (**2**) and 6-methylpinocembrin (**3**). Among the chalcones, 2′,4′-dihydroxychalcone (**6**) was the most abundant compound, and three dihydrochalcones were also isolated: 2′,4′-dihydroxydihydrochalcone (**4**), 2′,4′-dihydroxy-6′-methoxydihydrochalcone (**7**), and 2′-methoxy-4′-hydroxydihydrochalcone (**8**) (Figure 2).

The structure of compound **1** was consistent with 7-O-prenyl-pinocembrin. The ^1^H NMR spectrum showed the typical flavanone pattern, including two meta-coupled aromatic protons at δ 6.13 (H-8) and 6.08 (H-6), indicating substitution at C-7. Signals corresponding to a prenyl moiety were observed at δ 5.39 (H-2″), 4.57 (H-1″), 1.73 (Me-5″), and 1.68 (Me-4″), together with the corresponding ^13^C resonances at δ 65.1 (C-1″), 119.0 (C-2″), and 138.1 (C-3″). The downfield shift in C-7 (δ 166.7) and the presence of the benzylic methylene at δ 4.57 supported O-prenylation at C-7 rather than C-5. These data are consistent with a 7-O-prenylated pinocembrin structure. Compared to pinocembrin (**5**), compound **1** displayed characteristic signals of a prenyl substituent while retaining the meta-coupled H-6/H-8 pattern, confirming substitution at C-7.

The structure of compound **2** was consistent with 6,8-dimethylpinocembrin. The ^1^H NMR spectrum displayed two singlets integrating for three protons each at δ 1.96 and 1.94, indicative of two aromatic methyl groups, with corresponding ^13^C resonances at δ 8.8 and 8.1. The absence of aromatic proton signals in ring A confirmed substitution at both C-6 and C-8, consistent with a 6,8-dimethylated flavanone structure.

In the one-dimensional NMR spectra of compound **3**, signals consistent with the presence of a single, isolated methyl group appear: a singlet for three protons at 1.87 ppm in the ^1^H-NMR spectrum and a signal at 6.9 ppm in the ^13^C-NMR spectrum. Both signals correlate with each other in the HSQC spectrum. The position of this methyl group is clearly established by the HMBC spectrum, which shows a long-range correlation between the OH group at position 5 of ring A (δ = 12.39 ppm) and the adjacent carbon at position 6 (δ = 103.3 ppm), and this, in turn, with the protons of this CH_3_. In addition, the presence of H-8 as a singlet at δ 5.99 indicates that C-8 remains unsubstituted, thereby excluding methylation at C-8.

The higher *Rf* value observed for compound **4** relative to compound **8** suggests the presence of intramolecular hydrogen bonding, consistent with a chelated structure involving the 2′-hydroxyl group and the carbonyl function. Such chelation reduces the effective polarity of the molecule and consequently weakens its interaction with the polar silica stationary phase. A similar behavior was observed for compound **7** when compared to compound **8**. In contrast, compound **8** lacks this intramolecular hydrogen bond due to methoxylation at C-2′, resulting in increased effective polarity and stronger adsorption on silica.

### 3.2. Cytotoxic Activity

The cytotoxic activity of the isolated compounds was evaluated against HT-29 human colon adenocarcinoma and ACHN human renal carcinoma cell lines, and the results are summarized in Table 1.

In HT-29 cells, most compounds showed weak or no cytotoxic activity at the tested concentrations (IC_50_ > 100 µM), with the exception of 2′,4′-dihydroxychalcone (**6**), which exhibited the highest activity with an IC_50_ value of 13.31 ± 0.48 µM, and 2′,4′-dihydroxydihydrochalcone (**4**), which showed moderate activity (IC_50_ = 62.23 ± 1.06 µM). Compound (**8**) displayed low cytotoxicity (IC_50_ = 75.69 ± 0.74 µM), whereas pinocembrin (**5**), its prenylated derivative (**1**), and the C-methylated flavanones (**2**, **3**) were inactive (IC_50_ > 100 µM).

In contrast, ACHN cells were more sensitive to several of the tested compounds. The highest cytotoxicity was again observed for 2′,4′-dihydroxychalcone (**6**), with an IC_50_ value of 9.43 ± 0.34 µM. The dihydrochalcones (**4**), (**7**), and (**8**) showed moderate activity, with IC_50_ values of 54.68 ± 1.62, 44.00 ± 0.36, and 65.51 ± 0.83 µM, respectively. Among the flavanones, pinocembrin (**5**) displayed weak activity (IC_50_ = 91.28 ± 3.03 µM), while its C-methylated derivatives showed increased cytotoxicity, with IC_50_ values of 64.36 ± 0.53 µM for 6-methylpinocembrin (**3**) and 28.74 ± 0.35 µM for 6,8-dimethylpinocembrin (**2**). The prenylated derivative 7-O-prenyl-pinocembrin (**1**) was inactive in both cell lines (IC_50_ > 100 µM).

## 4. Discussion

The predominance of 2′,4′-dihydroxychalcone (**6**) and the dihydrochalcones (**8**) and (**4**), together with the comparatively low abundance of flavanones, suggests that in *C. album* the main metabolic flux within the flavonoid pathway is directed toward chalcone accumulation and subsequent reduction, whereas chalcone isomerization to flavanones may represent a secondary branch. This biosynthetic pattern, in which chalcones and dihydrochalcones accumulate as major products rather than transient intermediates, is consistent with previous reports describing limited chalcone isomerase activity and enhanced chalcone reductase-mediated flux in certain plant systems [23]. Moreover, this profile may reflect a relatively conservative metabolic strategy favoring the accumulation of structurally simple intermediates over extensive downstream diversification. The leaves of this species represent a distinctive flavonoid profile that has not been previously described for this species.

From a taxonomic perspective, these phytochemical features are also consistent with the evolutionary relationships within the Ericaceae lineage. Historically, *Corema*, *Empetrum*, and *Ceratiola* were treated as members of Empetraceae; however, molecular phylogenetic analyses based on nuclear ITS and chloroplast *matK* sequences demonstrated that Empetraceae is nested within Ericaceae [24], a treatment formally adopted in the APG II classification [25] and maintained in subsequent updates [26]. Within this phylogenetic framework, the phytochemical data obtained in this work are consistent with the close evolutionary relationships among these genera. The predominance of chalcones, dihydrochalcones, and simple flavanones in *C. album* parallels the phenolic profiles previously reported for *Empetrum nigrum* and, to a lesser extent, for *Ceratiola ericoides*. Although phytochemical similarity cannot substitute for molecular phylogenetic evidence, the shared occurrence of these lipophilic flavonoid derivatives provides additional chemotaxonomic support for the close affinity among these genera within the clade of Ericaceae.

To the best of our knowledge, this is the first detailed phytochemical investigation of *C. album* leaves focused on flavonoid constituents together with the evaluation of their cytotoxic activity in the ACHN renal carcinoma cell line.

The present study demonstrated that 2′,4′-dihydroxychalcone exhibited higher cytotoxicity than its dihydrochalcone analogue and other natural derivatives in the two tested cell lines. This enhanced activity is likely associated with the presence of the α,β-unsaturated system, which increases electrophilicity and enables chalcones to act as Michael acceptors, facilitating covalent interactions with cellular nucleophiles, particularly thiol groups in proteins and glutathione (GSH), the major intracellular redox buffer [27,28]. Such interactions may lead to depletion of GSH and modification of redox-sensitive proteins, thereby altering intracellular redox signaling pathways that regulate critical cellular processes, including DNA synthesis, enzyme activity, selective gene expression, and cell cycle progression [29]. Additionally, interactions with thiol-containing biomolecules may trigger apoptotic pathways [28].

Disruption of cellular thiol homeostasis may also contribute to mitochondrial dysfunction. Mitochondria represent a major site of reactive oxygen species (ROS) generation, and xenobiotics capable of modifying mitochondrial activity may stimulate ROS production [30]. Increased levels of ROS, including superoxide, hydroxyl radicals, and hydrogen peroxide, combined with depletion of antioxidant defenses such as GSH, can lead to oxidative stress and damage to cellular components. These processes are frequently associated with the activation of apoptosis through redox-sensitive signaling pathways and mitochondrial permeability transition mechanisms [31]. Under cellular conditions, chalcones may further alter thiol status through enzyme-catalyzed conjugation reactions with GSH or through oxidation of reduced thiols to the corresponding disulfides [32].

Such mechanistic effects may partly explain the higher cytotoxic activity of 2′,4′-dihydroxychalcone compared to its dihydrochalcone analogue and other natural derivatives isolated in this study, highlighting the importance of the α,β-unsaturated moiety in enhancing the cytotoxic potential of chalcone-based compounds. However, the electrophilic character of α,β-unsaturated carbonyl compounds may also contribute to a certain degree of nonspecific toxicity through broad reactivity with cellular nucleophiles, a well-recognized property of electrophilic Michael acceptors that can interact with multiple thiol-containing biomolecules. These observations are consistent with previous reports indicating that α,β-unsaturated chalcones generally exhibit greater cytotoxicity than their saturated dihydrochalcone counterparts [33,34].

Previous studies have evaluated the cytotoxic activity of 2′,4′-dihydroxychalcone and several oxyalkylated derivatives against different cancer cell lines, including HT-29 (human colorectal adenocarcinoma), MCF-7 (human breast adenocarcinoma), and PC-3 (human prostate adenocarcinoma), as well as against the non-tumoral epithelial cell line CCD 841 CoN. In these studies, 2′,4′-dihydroxychalcone showed relatively weak cytotoxic activity, with IC_50_ values around 100 µM in the tested tumor cell lines. Among the evaluated derivatives, only one compound (a geranyl-substituted analogue) displayed cytotoxicity comparable to that observed in the present work (IC_50_ ≈ 9.43 µM), whereas most derivatives exhibited moderate activity. Compounds displaying IC_50_ values in the low micromolar range are generally considered promising starting points for further optimization in anticancer drug discovery [35]. In comparison, some of the chalcones and dihydrochalcones isolated from *C. album*, including 2′,4′-dihydroxydihydrochalcone, 2′,4′-dihydroxy-6′-methoxydihydrochalcone, and 2′-methoxy-4′-hydroxydihydrochalcone, showed IC_50_ values of 64.36, 44.00, and 65.51 µM against the ACHN cell line, respectively. These results suggest a differential cytotoxic response of chalcone derivatives depending on the cellular model, which is consistent with previously reported selectivity indices below 1 for 2′,4′-dihydroxychalcone [36].

The cytotoxic activity observed in the ACHN cell line is particularly noteworthy from a pharmacological perspective. Renal carcinoma cells, such as the ACHN cell line, which is widely used as a model of renal cell carcinoma, are well known for their intrinsic resistance to many conventional chemotherapeutic agents, which contributes to the generally poor response of this tumor type to classical cytotoxic chemotherapy [37]. In this context, the activity displayed by the chalcone derivatives isolated from *C. album* becomes especially relevant. Although previous studies reported selectivity indices suggesting preferential toxicity toward tumor cells for related chalcones, the absence of a non-tumoral cell model in the present study prevents confirmation of this behavior under our experimental conditions. Future studies should therefore include parallel evaluation in non-tumoral cell models in order to determine selectivity indices and better assess the therapeutic relevance of these compounds. In addition, further experiments aimed at elucidating the underlying mechanisms of cytotoxicity, such as analyses of apoptosis induction, oxidative stress, and redox imbalance, would help to clarify the biological effects of these chalcone derivatives.

The isolation of pinocembrin and its structurally modified derivatives, including methylated and prenylated analogues, represents a noteworthy aspect of the phytochemical profile of *C. album* and expands the known chemical diversity of this species. Pinocembrin (5,7-dihydroxyflavanone) is a relatively simple flavanone widely distributed in plants, propolis, and honey, and has attracted considerable pharmacological interest due to its diverse biological activities, including antioxidant, anti-inflammatory, neuroprotective, and anticancer effects. Previous studies have reported that pinocembrin can induce cytotoxic effects in several cancer cell lines through mechanisms involving mitochondrial dysfunction, caspase activation, and modulation of signaling pathways such as PI3K/AKT and STAT3 (7). However, its therapeutic potential may be limited by low aqueous solubility and rapid metabolic conjugation, which reduce bioavailability [38]. In this context, naturally occurring derivatives such as those identified in *C. album* may represent useful templates for future structure–activity relationship studies [39].

From a preliminary structure–activity relationship perspective, the results obtained for the flavanone derivatives suggest that subtle structural modifications can significantly influence cytotoxic activity. In the present study, the dimethylated derivative showed the highest activity against the ACHN cell line, followed by the monomethylated analogue, whereas the parent compound displayed lower activity and the prenylated derivative was essentially inactive under the tested conditions. The increased activity observed for the methylated derivatives may be related to changes in physicochemical properties such as membrane permeability or molecular conformation introduced by methyl substitution on the flavanone core [40]. Conversely, although prenylation often enhances biological activity in flavonoids due to increased hydrophobicity and improved membrane interactions, the prenylated pinocembrin derivative isolated in this study did not exhibit significant cytotoxicity, highlighting that the biological consequences of prenyl substitution depend strongly on its position and the overall structural context of the molecule [41].

Beyond the biological activity observed, the phytochemical profile of *C. album* highlights the relevance of two complementary flavonoid scaffolds with potential pharmacological interest: chalcones and flavanones. In particular, the strong cytotoxicity displayed by 2′,4′-dihydroxychalcone reinforces the importance of α,β-unsaturated chalcone systems as promising frameworks for the development of novel anticancer agents. At the same time, the identification of methylated and prenylated derivatives of pinocembrin expands the known chemical diversity of naturally occurring flavanones in this species. Such structural modifications of the pinocembrin scaffold remain relatively underreported in many plant taxa and may reflect specific enzymatic tailoring processes within plant secondary metabolism. The occurrence of these derivatives is especially noteworthy in light of the increasing pharmacological interest in pinocembrin and the ongoing efforts aimed at generating analogues with improved physicochemical and biological properties [42]. In this context, the discovery of naturally occurring structural variants of both chalcones and pinocembrin derivatives in *C. album* provides valuable templates for future structure–activity studies and the development of new bioactive compounds.

## 5. Conclusions

This study demonstrates that *C. album* leaves are a rich source of phenolic compounds, predominantly chalcones, dihydrochalcones, and simple flavanones, reflecting a flavonoid biosynthetic profile oriented toward early pathway intermediates and reduced derivatives.

The cytotoxic evaluation revealed a preferential activity against ACHN renal carcinoma cells, a model characterized by intrinsic resistance to anticancer drugs, while limited effects were observed in HT-29 colon cancer cells. Among the chalcone-related structures evaluated, 2′,4′-dihydroxychalcone displayed the highest activity, showing greater cytotoxicity than its corresponding dihydrochalcone analogue and the other natural derivatives tested. This enhanced activity may be associated with the presence of the α, β-unsaturated system, which likely increases electrophilicity and may contribute to the cytotoxic potential of chalcone-based scaffolds.

In addition, the differences in activity observed among the pinocembrin derivatives suggest that subtle structural modifications, such as C-methylation, may significantly influence the biological properties of the flavanone scaffold. Taken together, these findings provide new insights into the flavonoid chemistry of *C. album* and highlight this species as a source of structurally diverse phenolic metabolites. Although the biological evaluation performed here remains preliminary, the observed cytotoxicity and the structural diversity of the isolated compounds suggest that this plant may represent a promising source of lead molecules for further pharmacological investigation. Future studies addressing broader biological screening and mechanistic analyses will be necessary to fully assess the therapeutic potential of these metabolites.

## Figures and Tables

**Table 1:** Cytotoxicity of isolated compounds **1**−**8** ^a^.

	IC_50_ (µM)	
Compound	HT-29	ACHN
7-O-prenyl-pinocembrin (**1**)	>100	>100
6,8-dimethylpinocembrin (**2**)	>100	28.74 ± 0.35
6-methylpinocembrin (**3**)	>100	64.36 ± 0.53
2′,4′-dihydroxydihydrochalcone (**4**)	62.23 ± 1.06	54.68 ± 1.62
Pinocembrin (**5**)	>100	91.28 ± 3.03
2′,4′-dihydroxychalcone (**6**)	13.31 ± 0.48	9.43 ± 0.34
2′,4′-dihydroxy-6′-methoxy-dihydrochalcone (**7**)	>100	44.00 ± 0.36
2′-methoxy-4′-hydroxydihydrochalcone (**8**)	75.69 ± 0.74	65.51 ± 0.83
Doxorubicin	11.42 ± 0.26	3.92 ± 0.11

^a^ Cell viability was measured using the MTT assay after 72 h of incubation. Data are expressed as the mean ± SD of three independent experiments.

## Data Availability

The data presented in this study are available in the article and Supplementary Materials.

## Supplementary Materials

The following supporting information can be downloaded at: https://www.mdpi.com/article/10.3390/biom16040509/s1, Figure S1: ^1^H NMR spectrum of compound **1** in DMSO-d_6_; Figure S2: ^13^C NMR spectrum of compound **1** in DMSO-d_6_; Figure S3: 2D COSY NMR spectrum of compound **1** in DMSO-d_6_; Figure S4: 2D HSQC NMR spectrum of compound **1** in DMSO-d_6_; Figure S5: 2D HMBC NMR spectrum of compound **1** in DMSO-d_6_; Figure S6: Mass spectrum of compound **1**; Figure S7: ^1^H NMR spectrum of compound **2** in DMSO-d_6_; Figure S8: ^13^C NMR spectrum of compound **2** in DMSO-d_6_; Figure S9: Mass spectrum of compound **2**; Figure S10: ^1^H NMR spectrum of compound **3** in DMSO-d_6_; Figure S11: ^13^C NMR spectrum of compound **3** in DMSO-d_6_; Figure S12: 2D COSY NMR spectrum of compound **3** in DMSO-d_6_; Figure S13: 2D HSQC NMR spectrum of compound **3** in DMSO-d_6_; Figure S14: 2D HMBC NMR spectrum of compound **3** in DMSO-d_6_; Figure S15: Mass spectrum of compound **3**; Figure S16: ^1^H NMR spectrum of compound **4** in DMSO-d_6_; Figure S17: ^13^C NMR spectrum of compound **4** in DMSO-d_6_; Figure S18: Mass spectrum of compound **4**; Figure S19: ^1^H NMR spectrum of compound **5** in DMSO-d_6_; Figure S20: ^13^C NMR spectrum of compound **5** in DMSO-d_6_; Figure S21: Mass spectrum of compound **5**; Figure S22: ^1^H NMR spectrum of compound **6** in DMSO-d_6_; Figure S23: ^13^C NMR spectrum of compound **6** in DMSO-d_6_; Figure S24: 2D COSY NMR spectrum of compound **6** in DMSO-d_6_; Figure S25: 2D HSQC NMR spectrum of compound **6** in DMSO-d_6_; Figure S26: Mass spectrum of compound **6**; Figure S27: ^1^H NMR spectrum of compound **7** in DMSO-d_6_; Figure S28: ^13^C NMR spectrum of compound **7** in DMSO-d_6_; Figure S29: Mass spectrum of compound **7**; Figure S30: ^1^H NMR spectrum of compound **8** in DMSO-d_6_; Figure S31: ^13^C NMR spectrum of compound **8** in DMSO-d_6_; Figure S32: 2D COSY NMR spectrum of compound **8** in DMSO-d_6_; Figure S33: 2D HSQC NMR spectrum of compound **8** in DMSO-d_6_; Figure S34: 2D HMBC NMR spectrum of compound **8** in DMSO-d_6_; Figure S35: Mass spectrum of compound **8**.
