# Supplementary material for: Cytotoxicity Evaluation of Chalcones and Flavanones from the Leaves of Corema album (L.) D. Don
Source: Biomolecules. 2026 Mar 28;16(4):509. doi: 10.3390/biom16040509 (PMC13113417; doi:10.3390/biom16040509)
Supplement: Supplementary file 1 [file biomolecules-16-00509-s001.zip › biomolecules-4192887-supplementary.pdf]

Supplementary material

Cytotoxicity Evaluation of Chalcones and Flavanones from the Leaves of *Corema album* (L.) D. Don

Antonio Canoyra[1], Nuria Acero[1], Dolores Muñoz-Mingarro[2], Antonio José León-González[3], José Luis Espartero[4], Carmen Martín-Cordero[3,*]

[1]Pharmaceutical and Health Science Department, Pharmacy Faculty, San Pablo-CEU University, CEU Universities, Urbanización Montepríncipe Boadilla del Monte, 28660 Madrid, Spain; a.canoyra@usp.ceu.es; nacemes@ceu.es

[2]Chemistry and Biochemistry Department, Pharmacy Faculty, San Pablo-CEU University, CEU Universities, Urbanización Montepríncipe, Boadilla del Monte, 28668 Madrid, Spain; dmumin@ceu.es

[3]Department of Pharmacology, Faculty of Pharmacy, University of Seville, C/P. García González, 2, 41012 Seville, Spain. ajleon@us.es ; carmenmc@us.es

[4]Department of Organic and Pharmaceutical Chemistry, Faculty of Pharmacy, University of Seville, 41012 Sevilla, Spain. jles@us.es

*Correspondence: carmenmc@us.es

Table of contents

Figure S1. ^{1}H NMR spectrum of compound **1**..3
Figure S2. ^{13}C NMR spectrum of compound **1** 4
Figure S3. 2D COSY NMR spectrum of compound **1** 5
Figure S4. 2D HSQC NMR spectrum of compound **1** 6
Figure S5. 2D HMBC NMR spectrum of compound **1** 7
Figure S6. MS spectrum of compound **1**..8
Figure S7. ^{1}H NMR spectrum of compound **2** 9
Figure S8. ^{13}C NMR spectrum of compound **2** 10
Figure S9. MS spectrum of compound **2**..11
Figure S10. ^{1}H NMR spectrum of compound **3**..12
Figure S11. ^{13}C NMR spectrum of compound **3** 13
Figure S12. 2D COSY NMR spectrum of compound **3** 14
Figure S13. 2D HSQC NMR spectrum of compound **3**...................................... 15
Figure S14. 2D HMBC NMR spectrum of compound **3** 16
Figure S15. MS spectrum of compound **3**..17
Figure S16. ^{1}H NMR spectrum of compound **4** 18
Figure S17. ^{13}C NMR spectrum of compound **4** 19
Figure S18. MS spectrum of compound **4**..20
Figure S19. ^{1}H NMR spectrum of compound **5** 21
Figure S20. ^{13}C NMR spectrum of compound **5** 22
Figure S21. MS spectrum of compound **5**..23
Figure S22. ^{1}H NMR spectrum of compound **6** 24
Figure S23. ^{13}C NMR spectrum of compound **6** 25
Figure S24. 2D COSY NMR spectrum of compound **6** 26
Figure S25. 2D HSQC NMR spectrum of compound **6** 27
Figure S26. MS spectrum of compound **6**... 28
Figure S27. ^{1}H NMR spectrum of compound **7** 29
Figure S28. ^{13}C NMR spectrum of compound **7** 30
Figure S29. MS spectrum of compound **7**..31
Figure S30. ^{1}H NMR spectrum of compound **8**...32
Figure S31. ^{13}C NMR spectrum of compound **8** 33
Figure S32. 2D COSY NMR spectrum of compound 8 34
Figure S33. 2D HSQC NMR spectrum of compound **8** 35
Figure S34. 2D HMBC NMR spectrum of compound **8** 36
Figure S35. MS spectrum of compound **8**...37

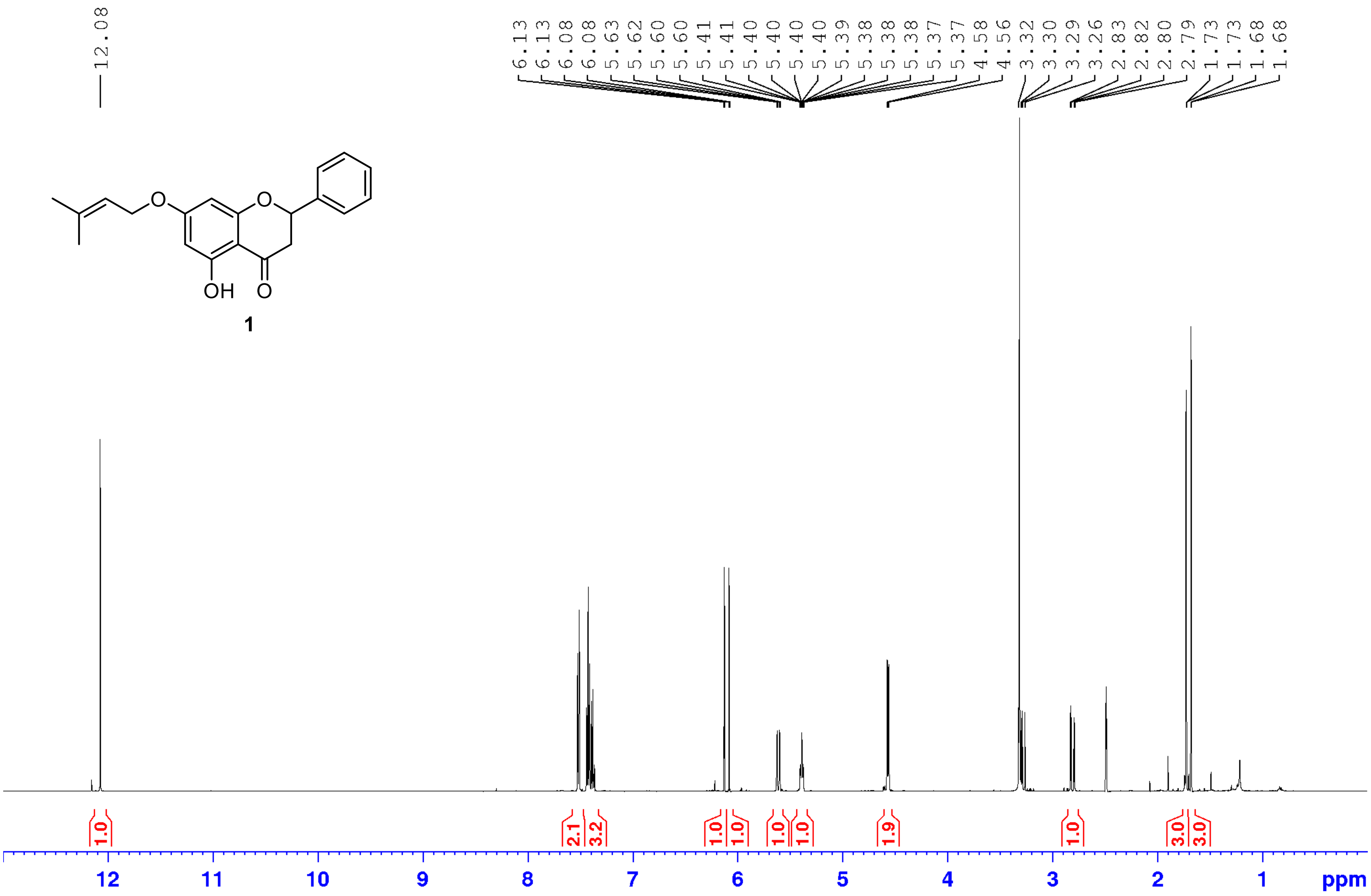

Figure S1. ^{1}H NMR spectrum of compound **1** in DMSO-d_6

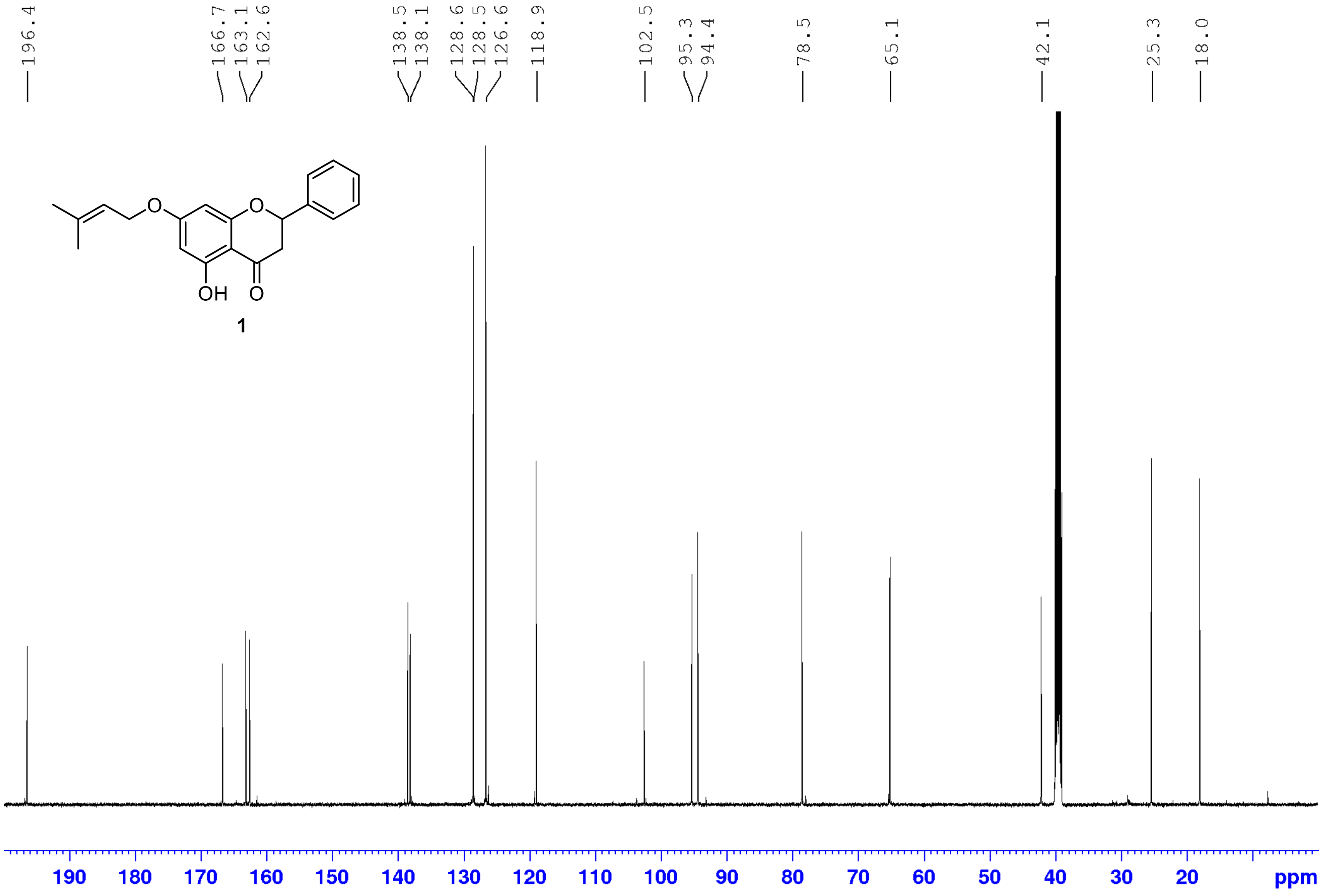

Figure S2. ^{13}C NMR spectrum of compound **1** in DMSO-d_6

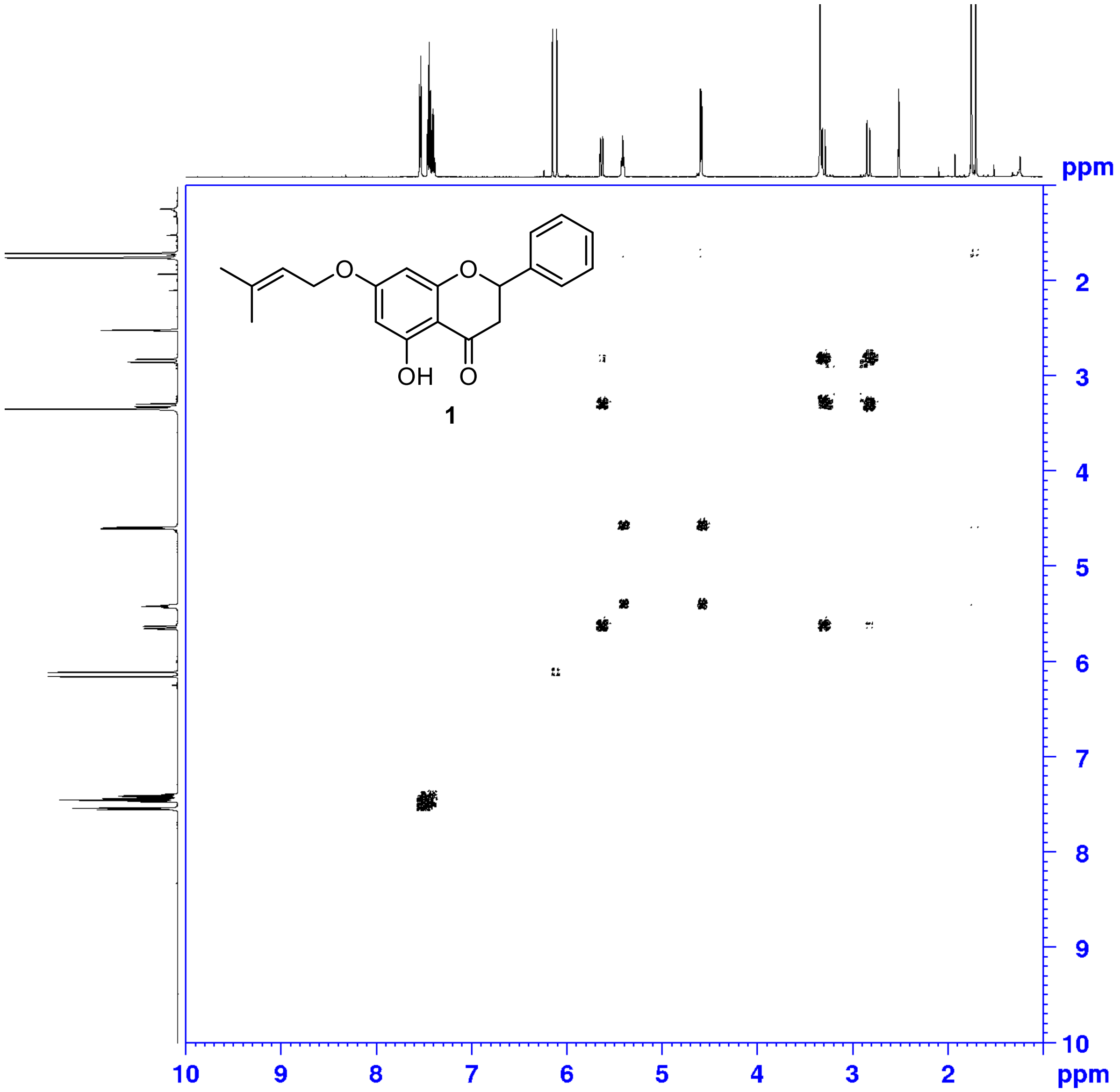

Figure S3. 2D COSY NMR spectrum of compound **1** in DMSO-d_6

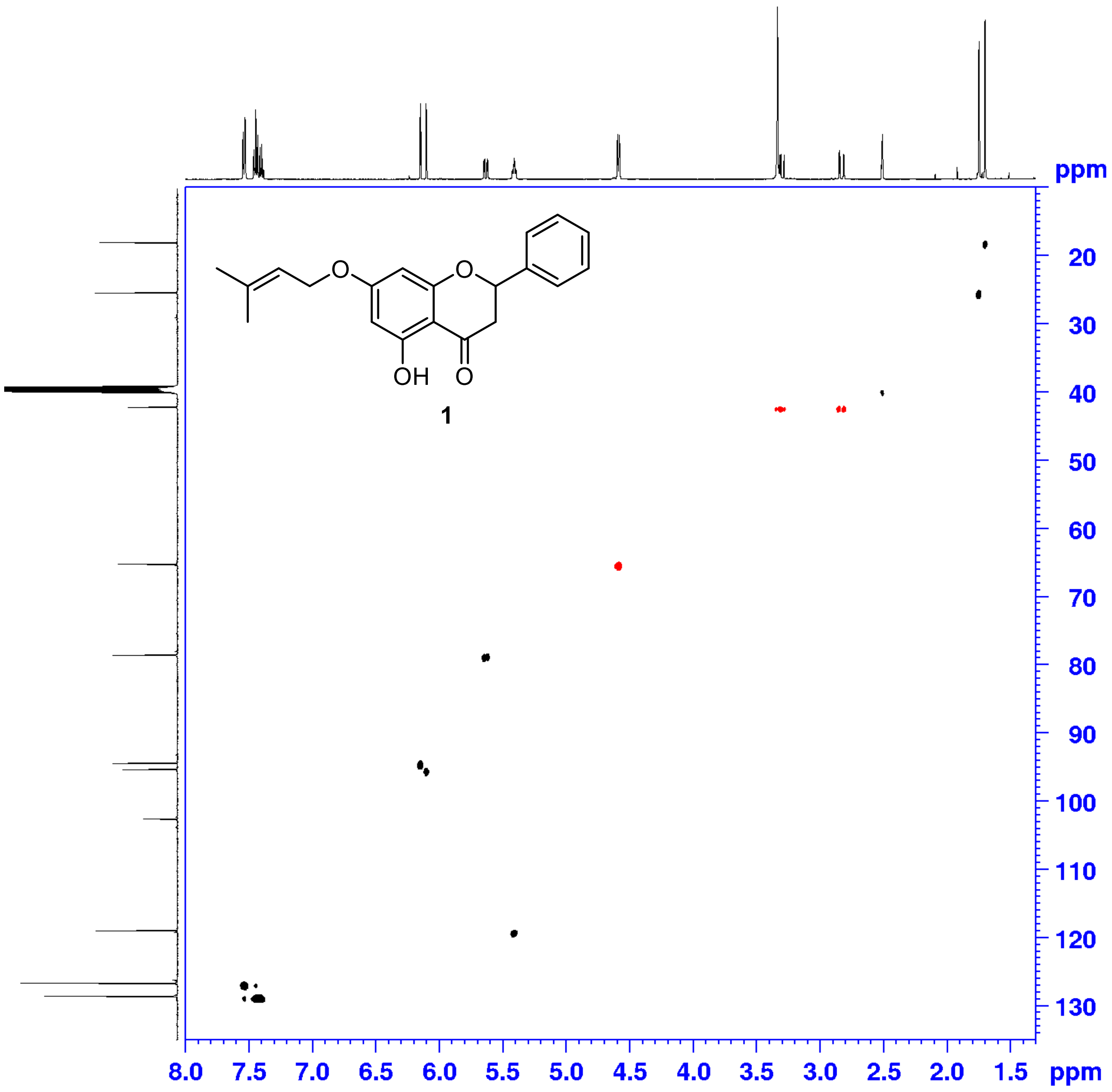

Figure S4. 2D HSQC NMR spectrum of compound **1** in DMSO-d_6

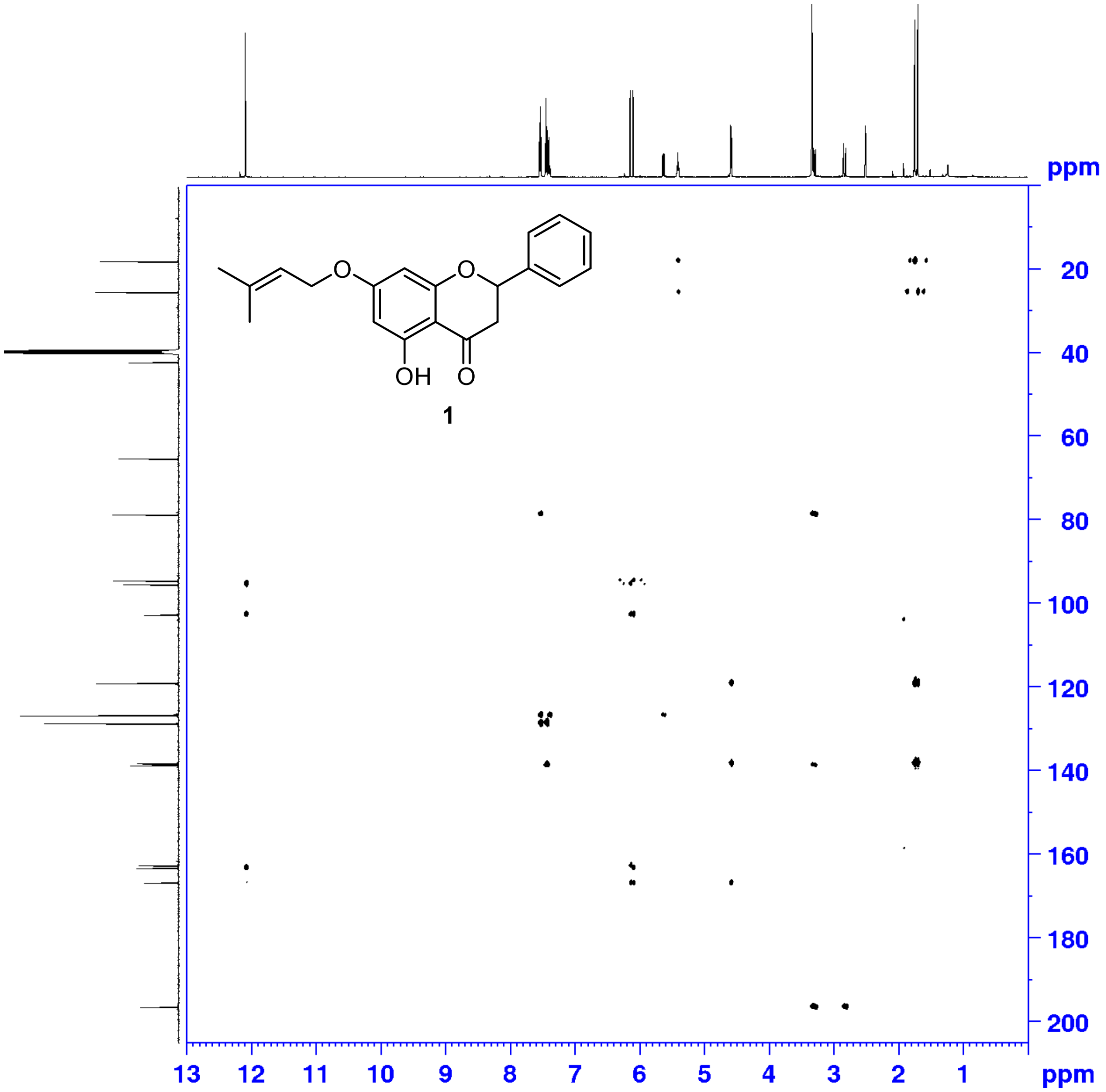

Figure S5. 2D HMBC NMR spectrum of compound **1** in DMSO-d_6

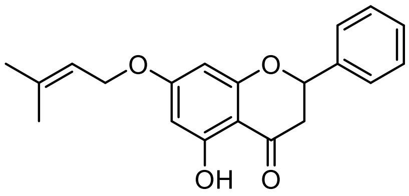

1

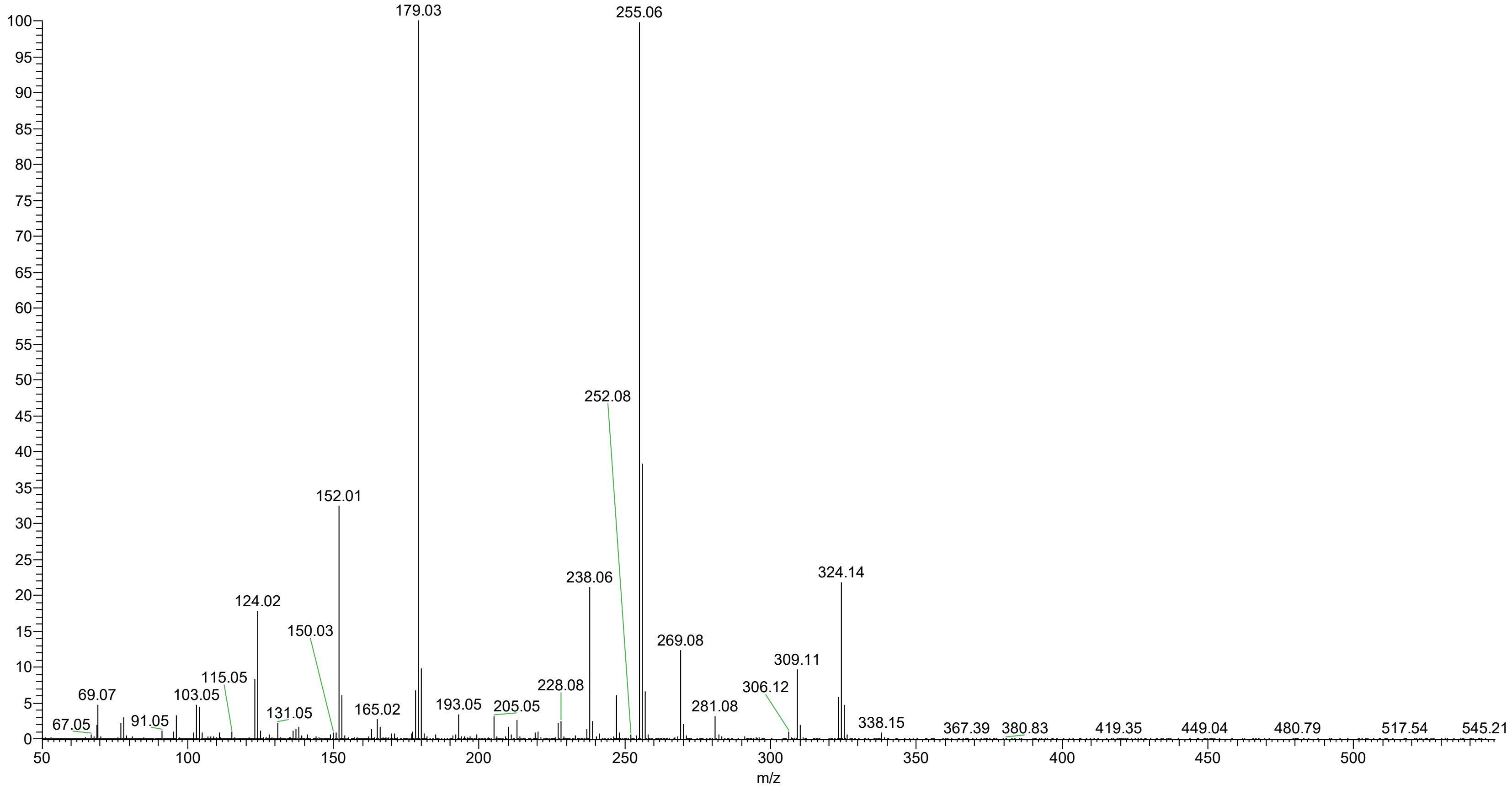

Figure S6. Mass spectrum of compound 1

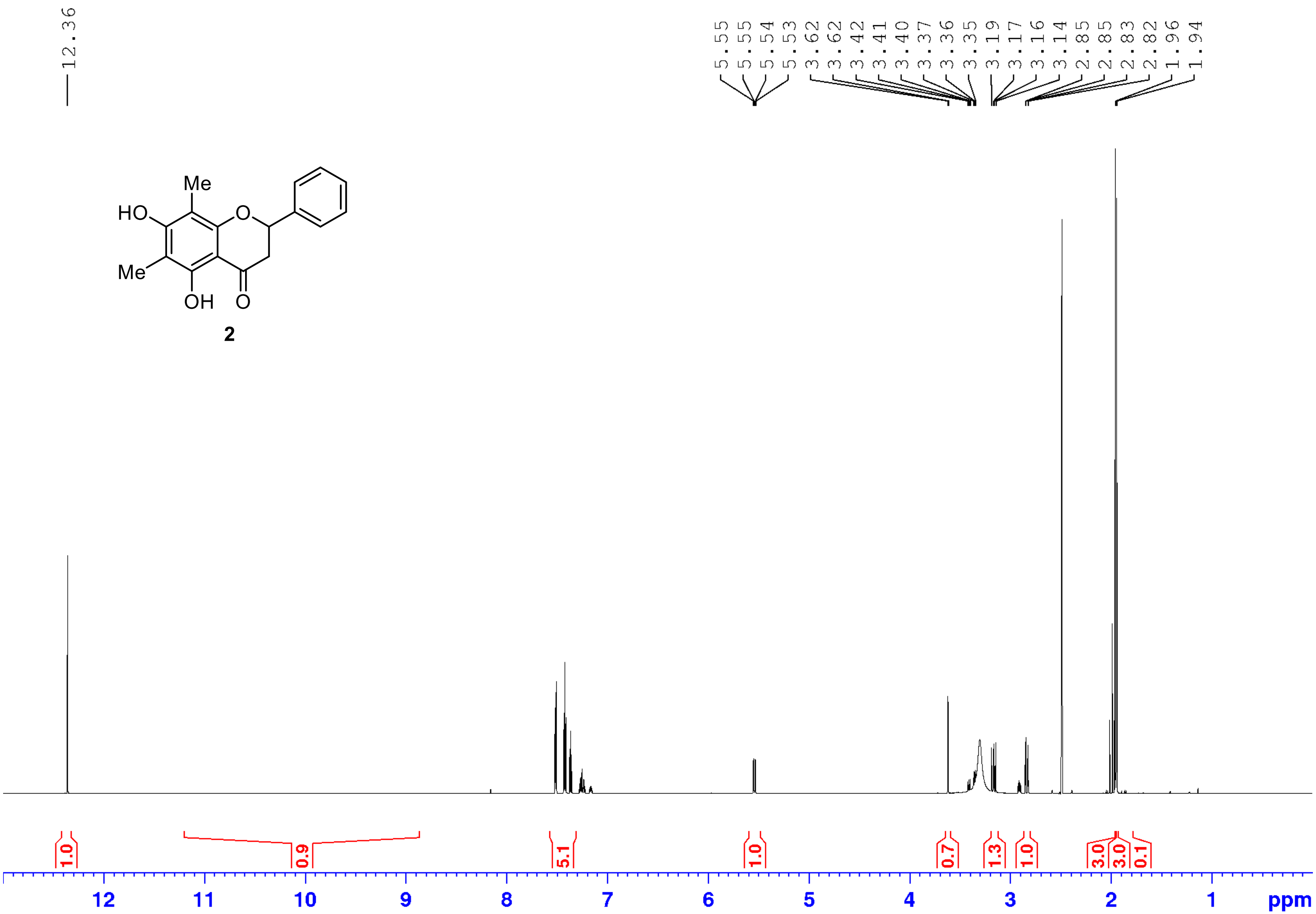

Figure S7. ^{1}H NMR spectrum of compound **2** in DMSO-d_6

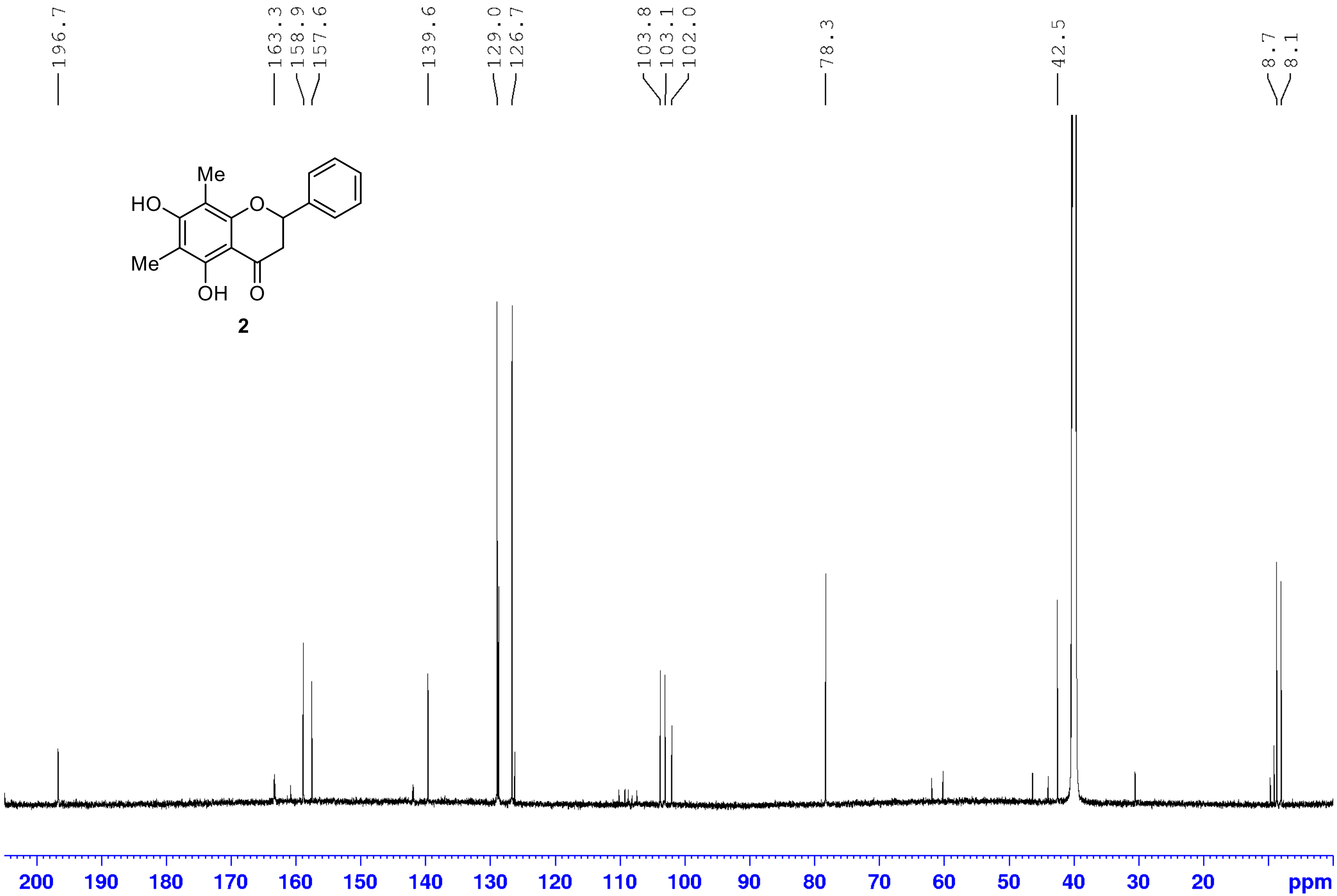

Figure S8. ^{13}C NMR spectrum of compound **2** in DMSO-d_6

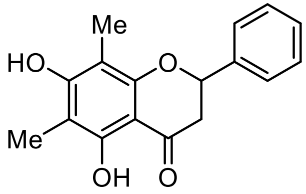

2

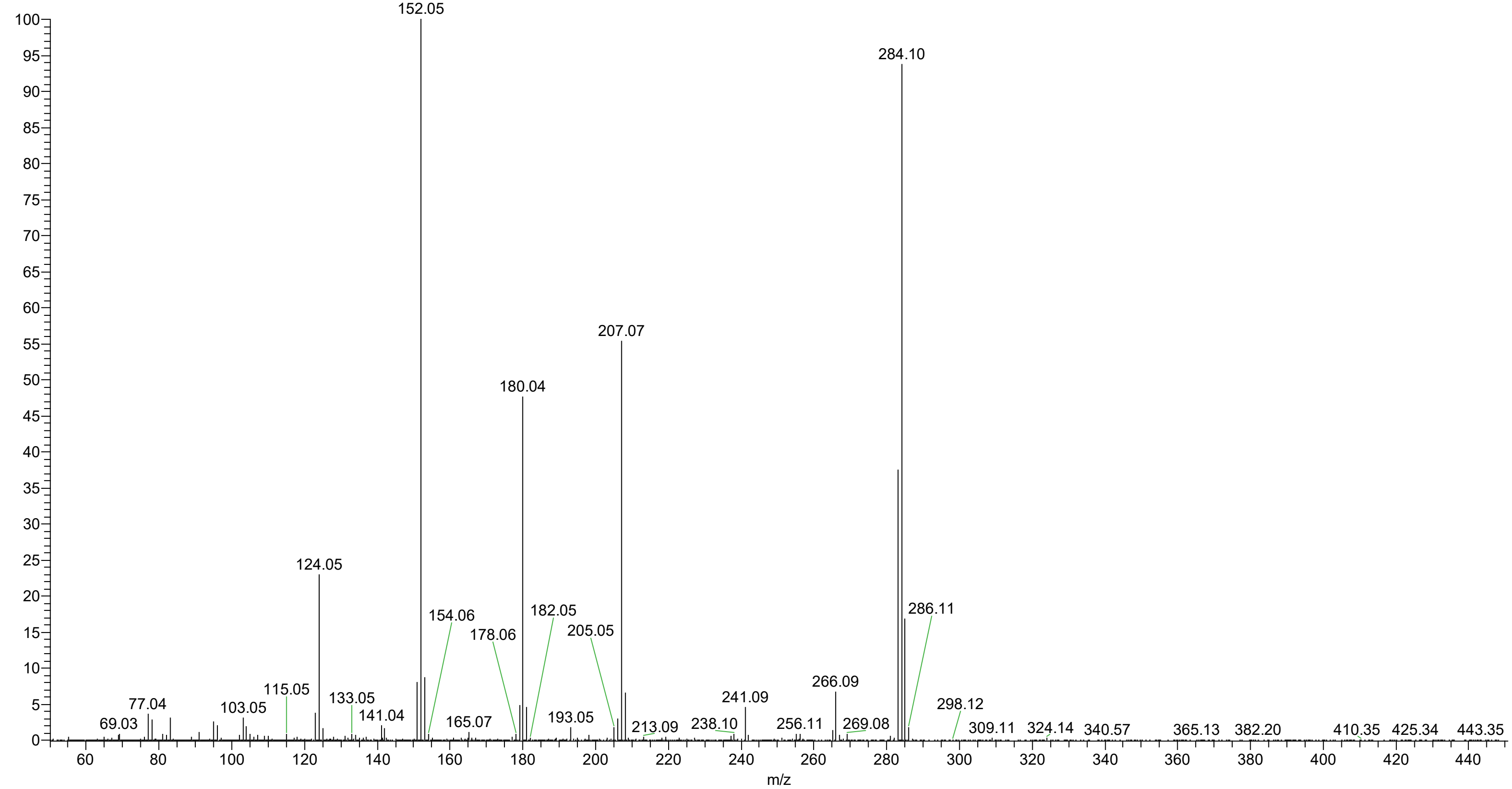

Figure S9. Mass spectrum of compound 2

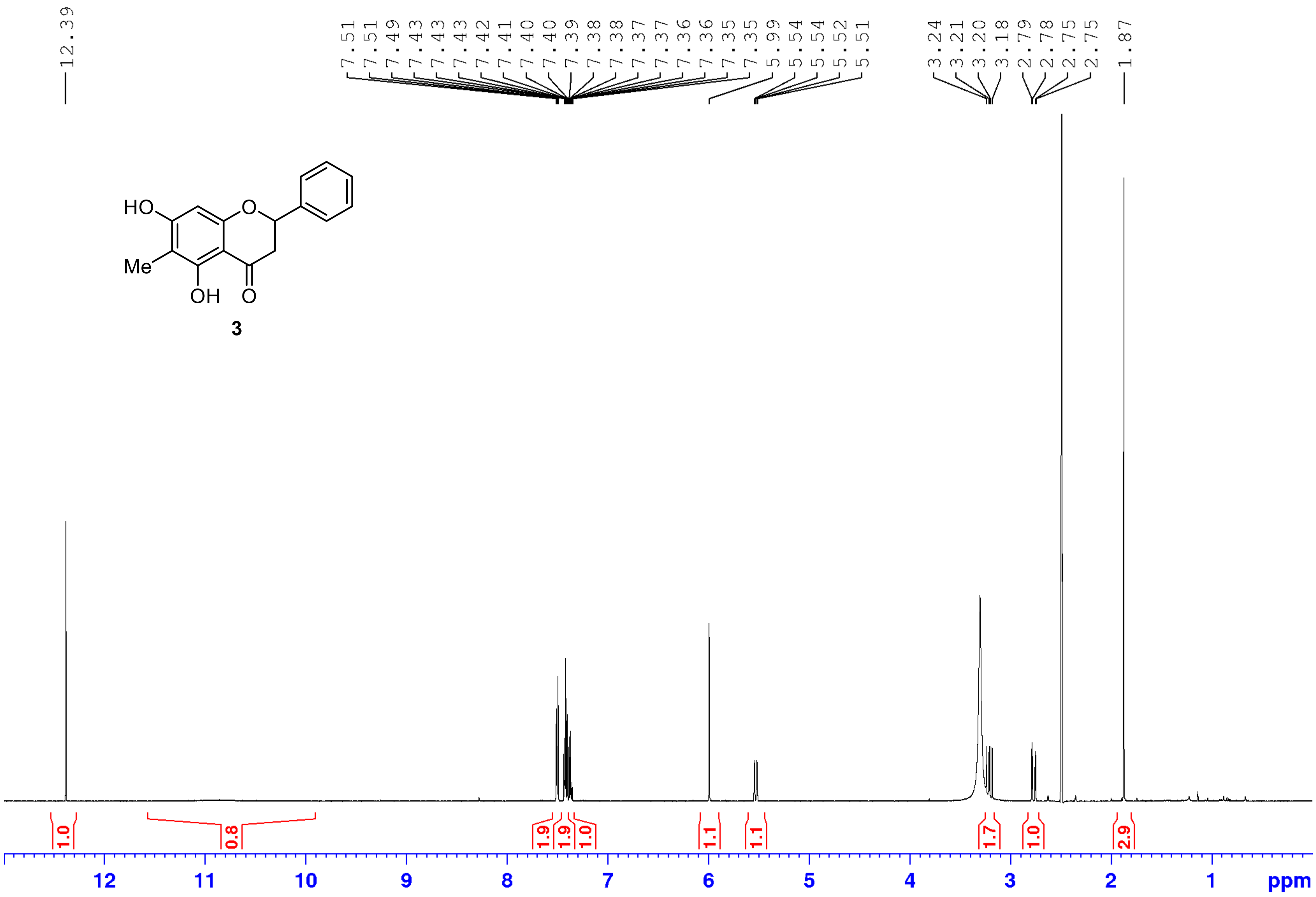

Figure S10. ^{1}H NMR spectrum of compound **3** in DMSO-d_6

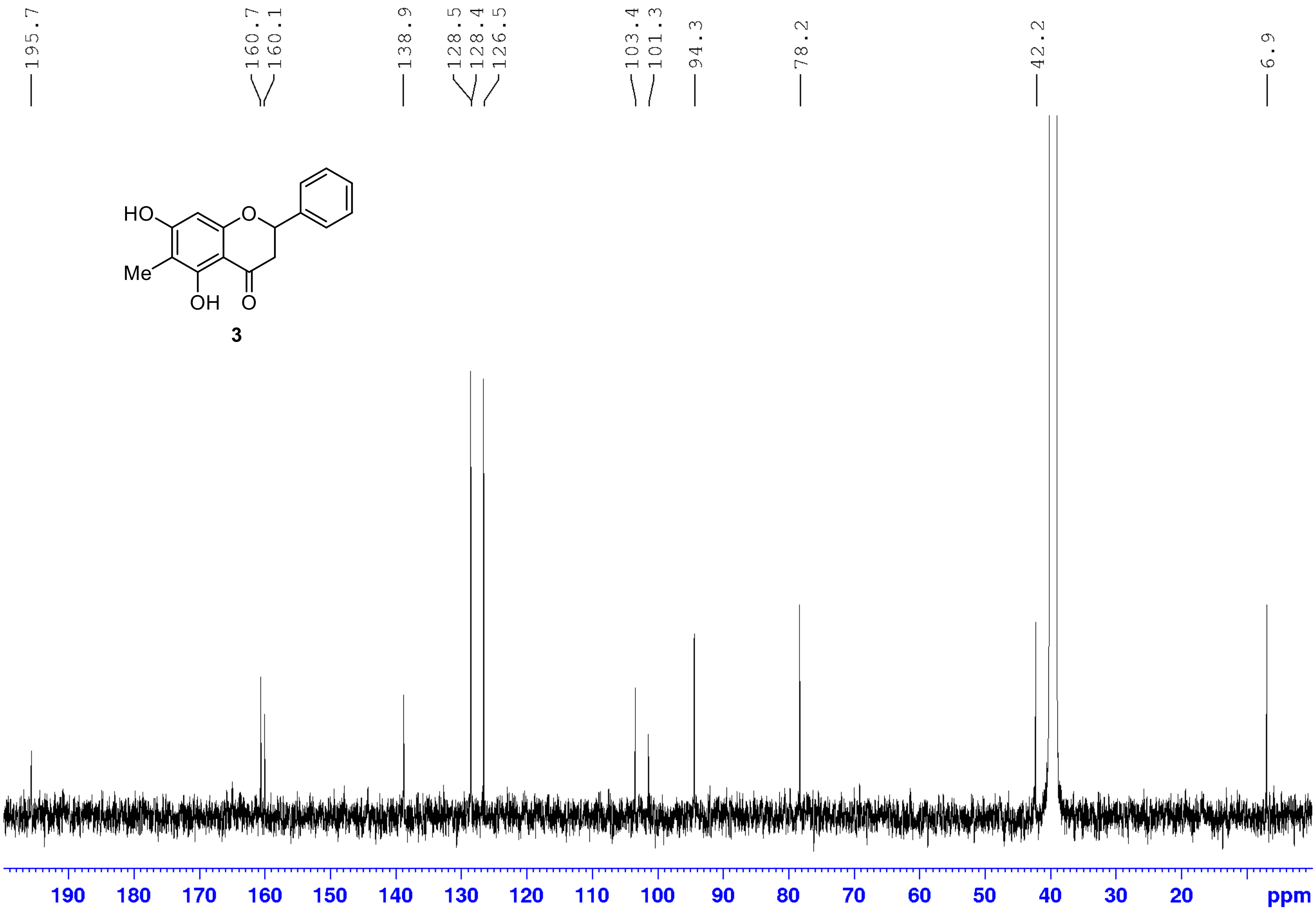

Figure S11. ^{13}C NMR spectrum of compound **3** in DMSO-d_6

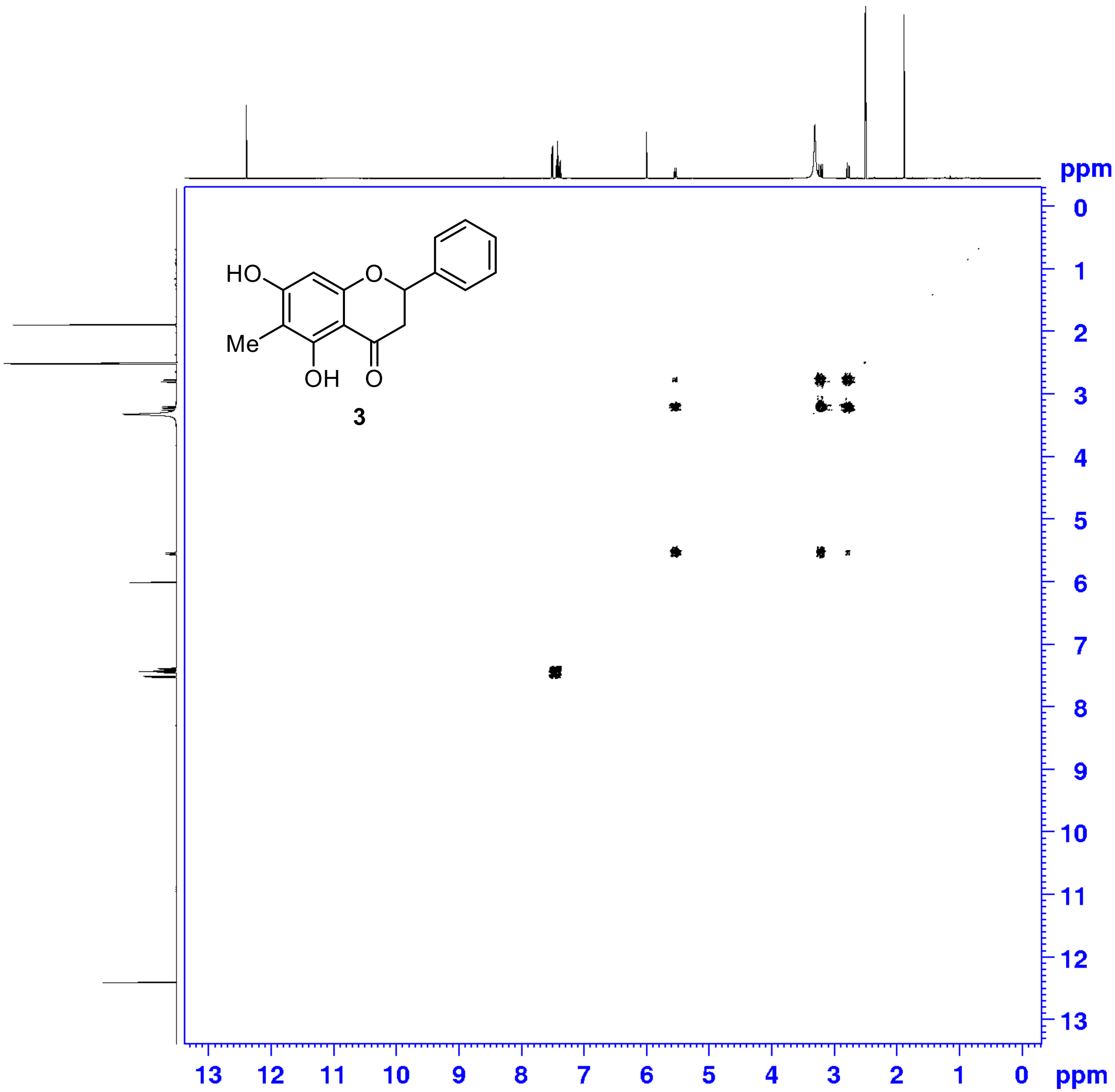

Figure S12. 2D COSY NMR spectrum of compound **3** in DMSO-d_6

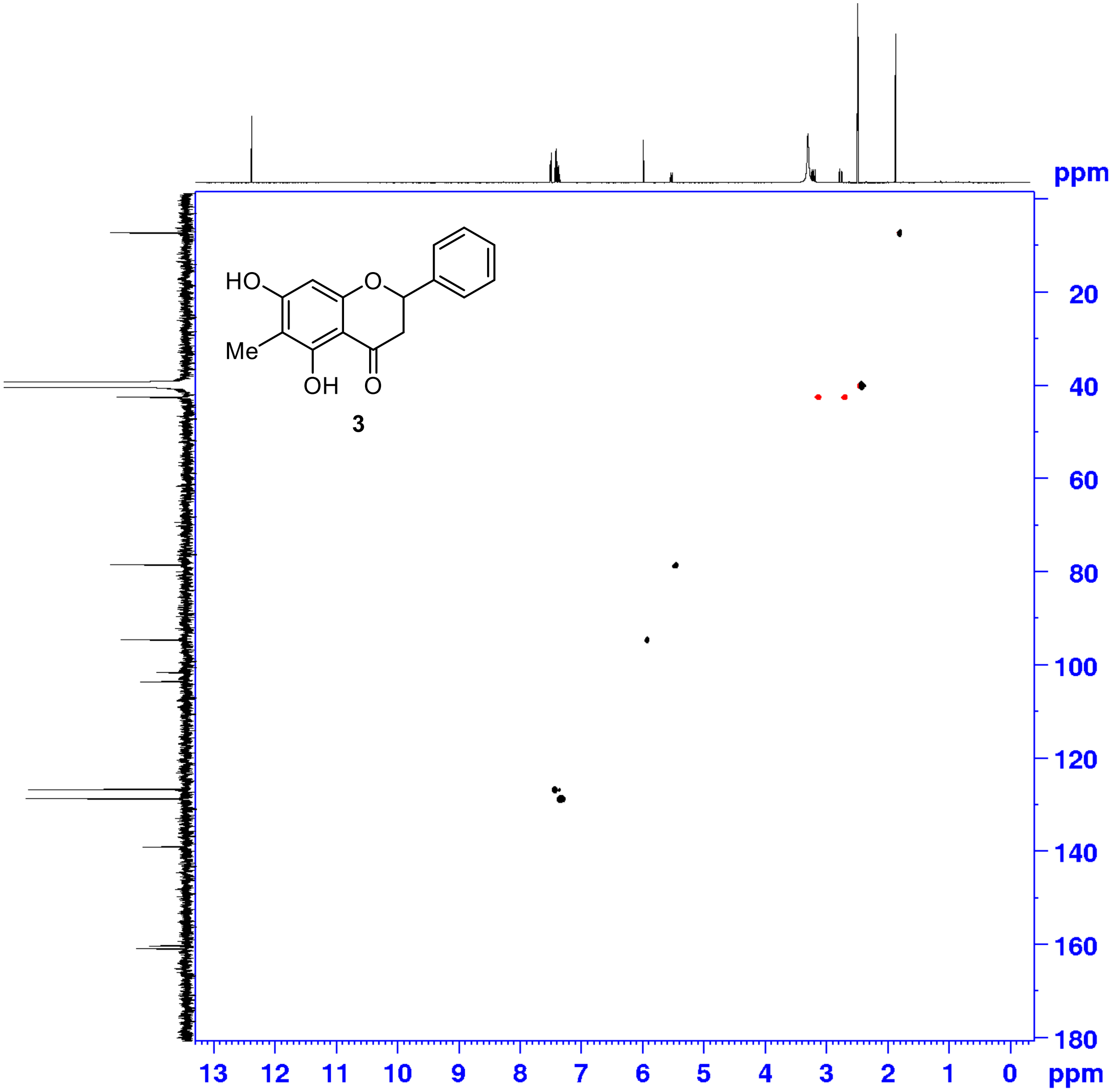

Figure S13. 2D HSQC NMR spectrum of compound **3** in DMSO-d_6

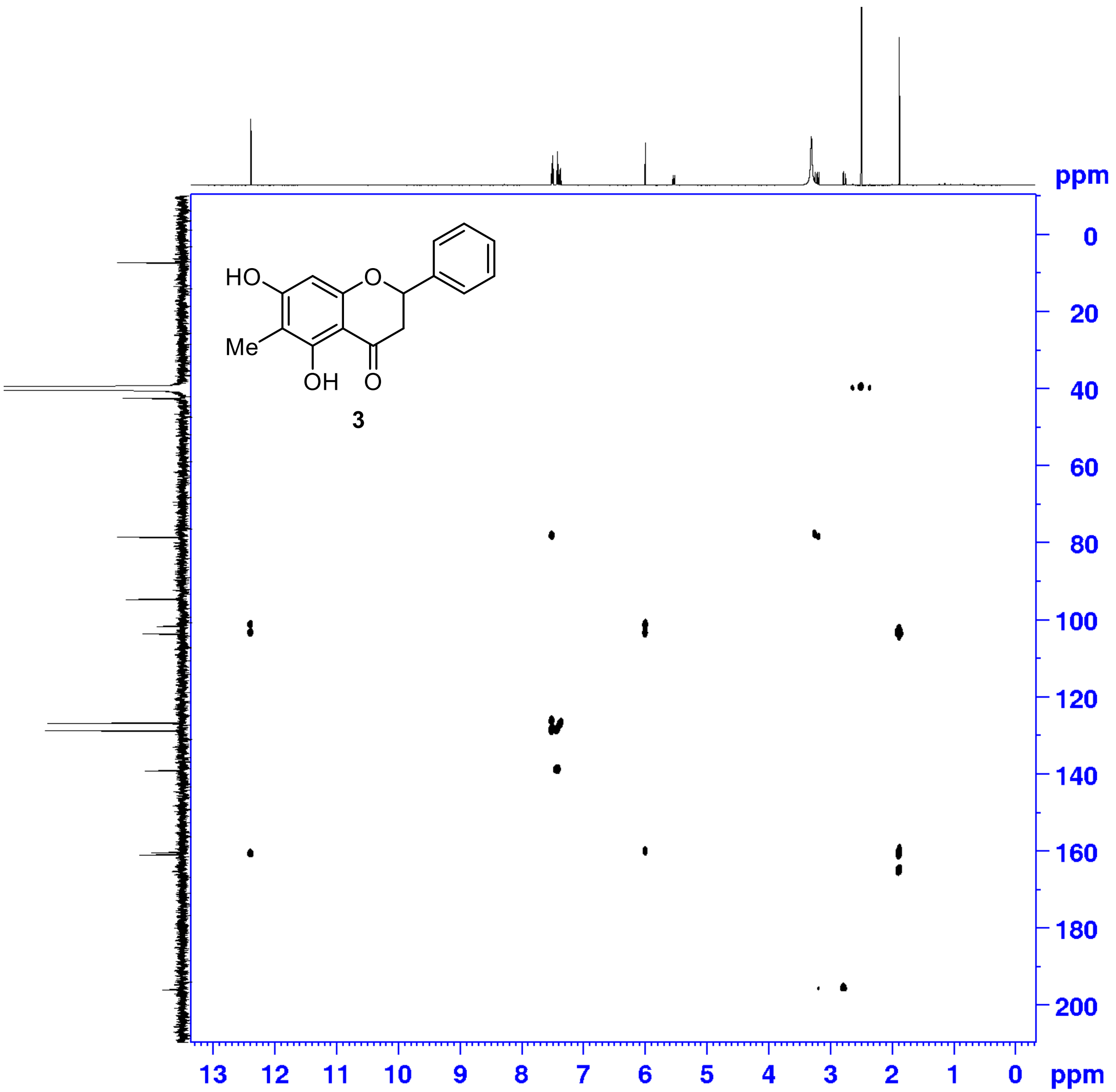

Figure S14. 2D HMBC NMR spectrum of compound **3** in DMSO-d_6

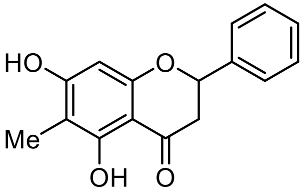

3

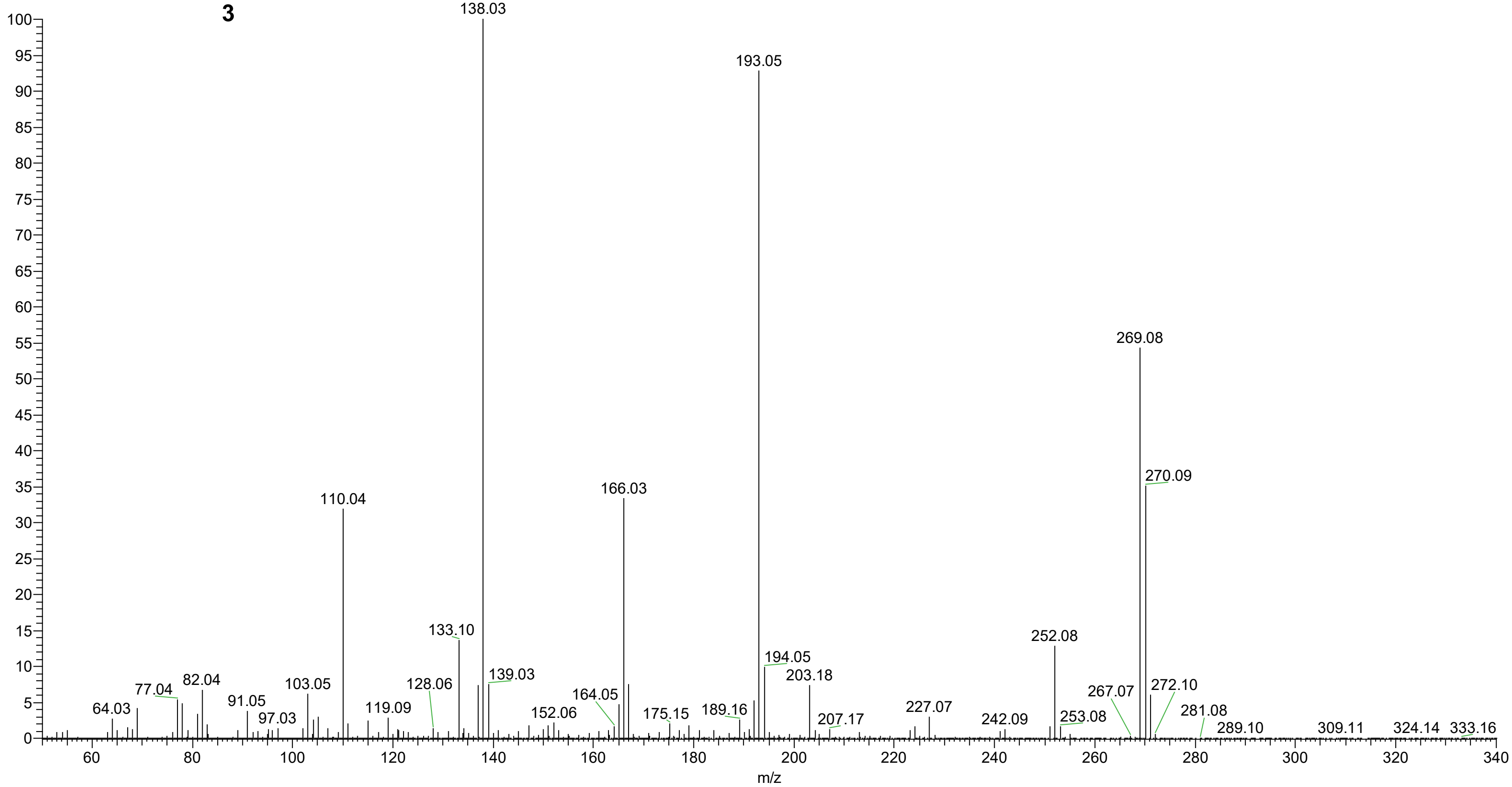

Figure S15. Mass spectrum of compound 3

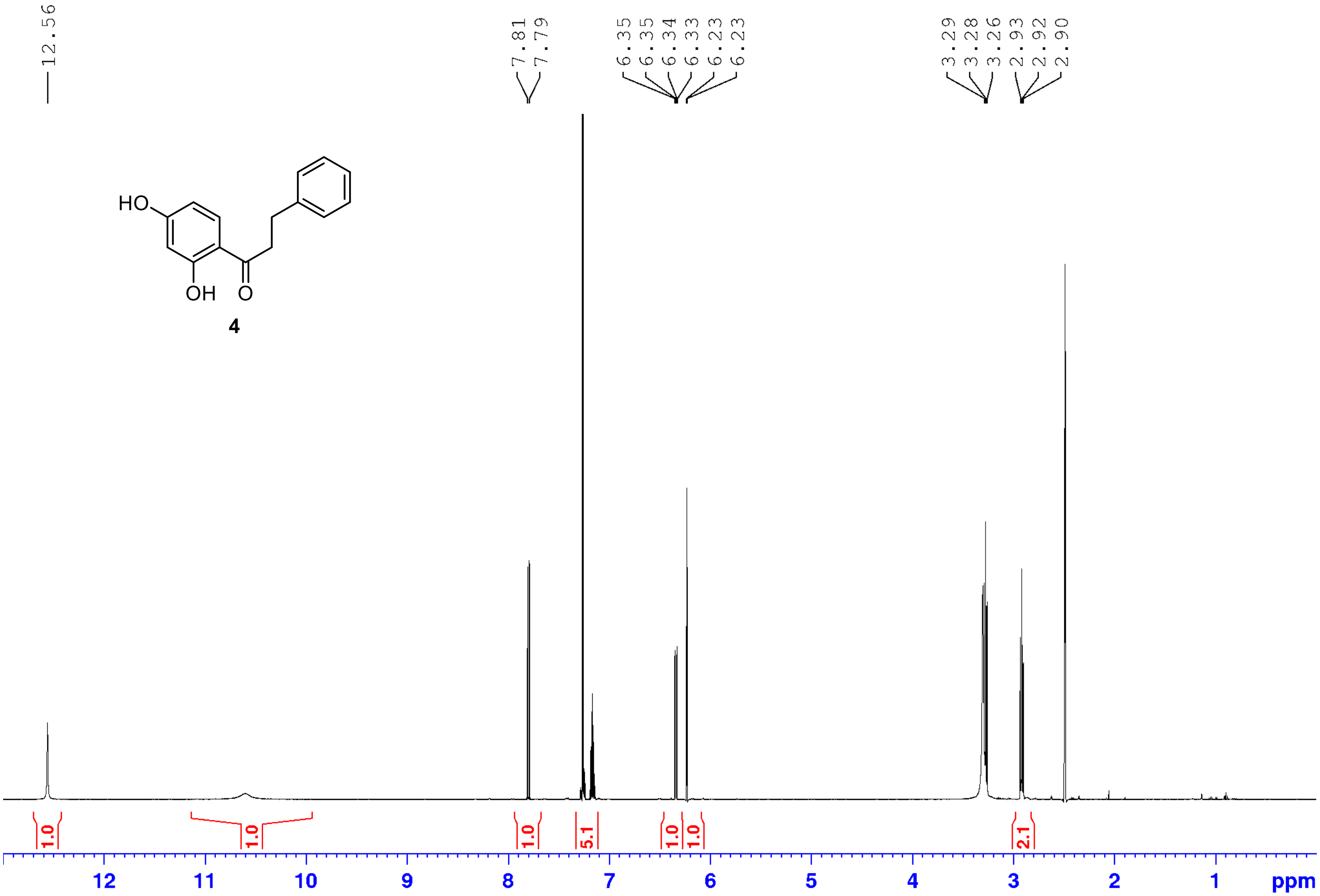

Figure S16. ^{1}H NMR spectrum of compound **4** in DMSO-d_6

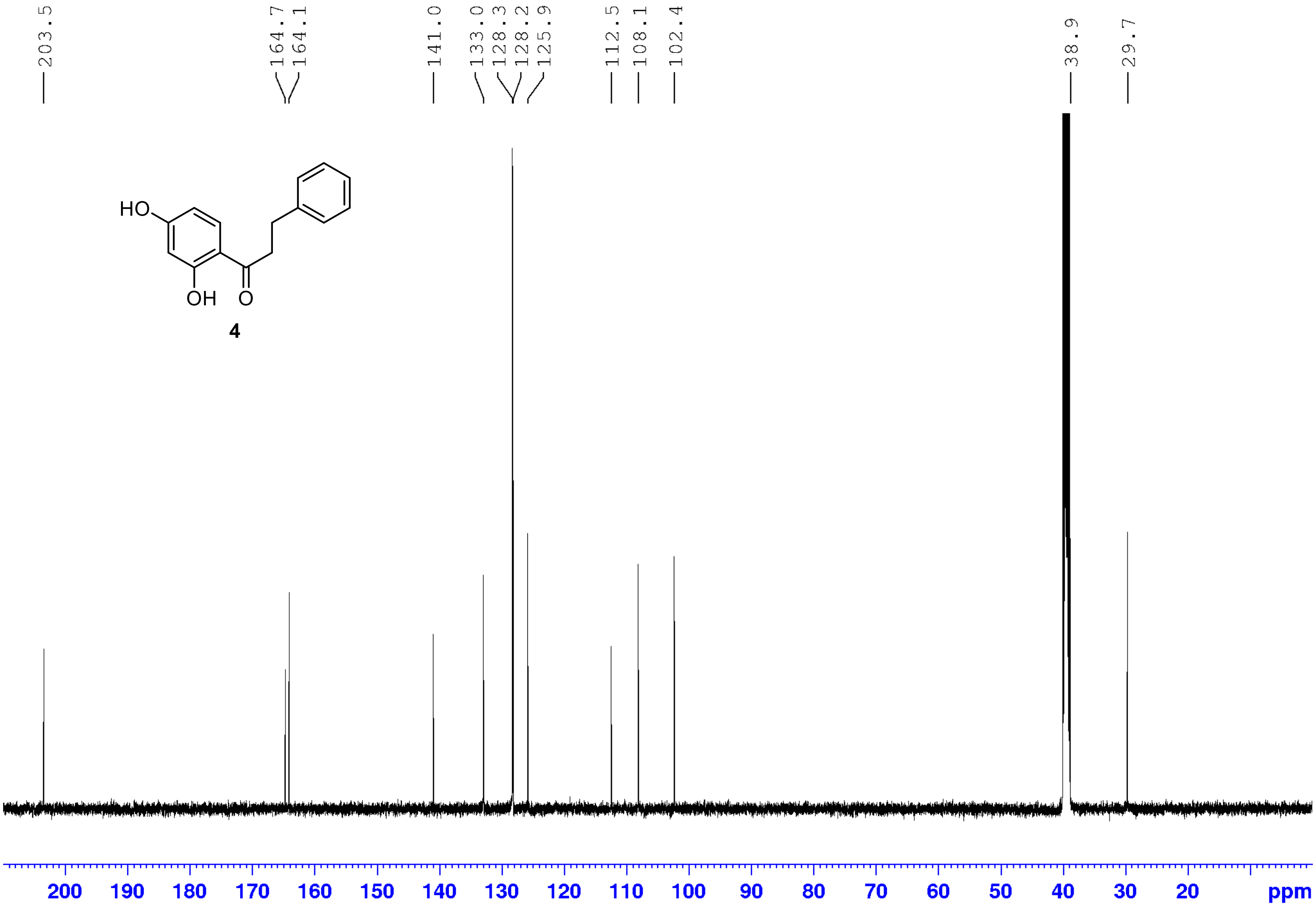

Figure S17. ^{13}C NMR spectrum of compound **4** in DMSO-d_6

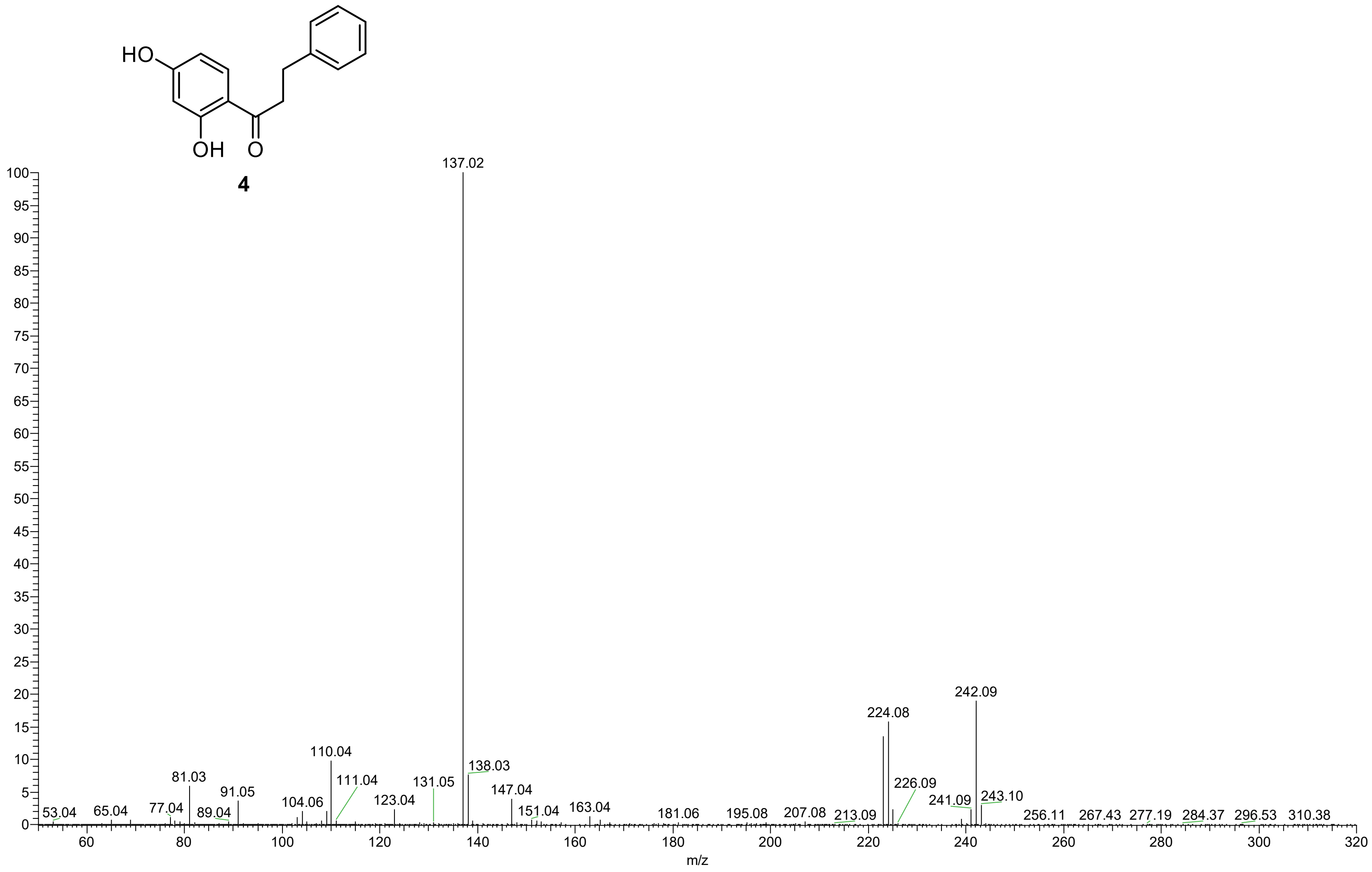

Figure S18. Mass spectrum of compound 4

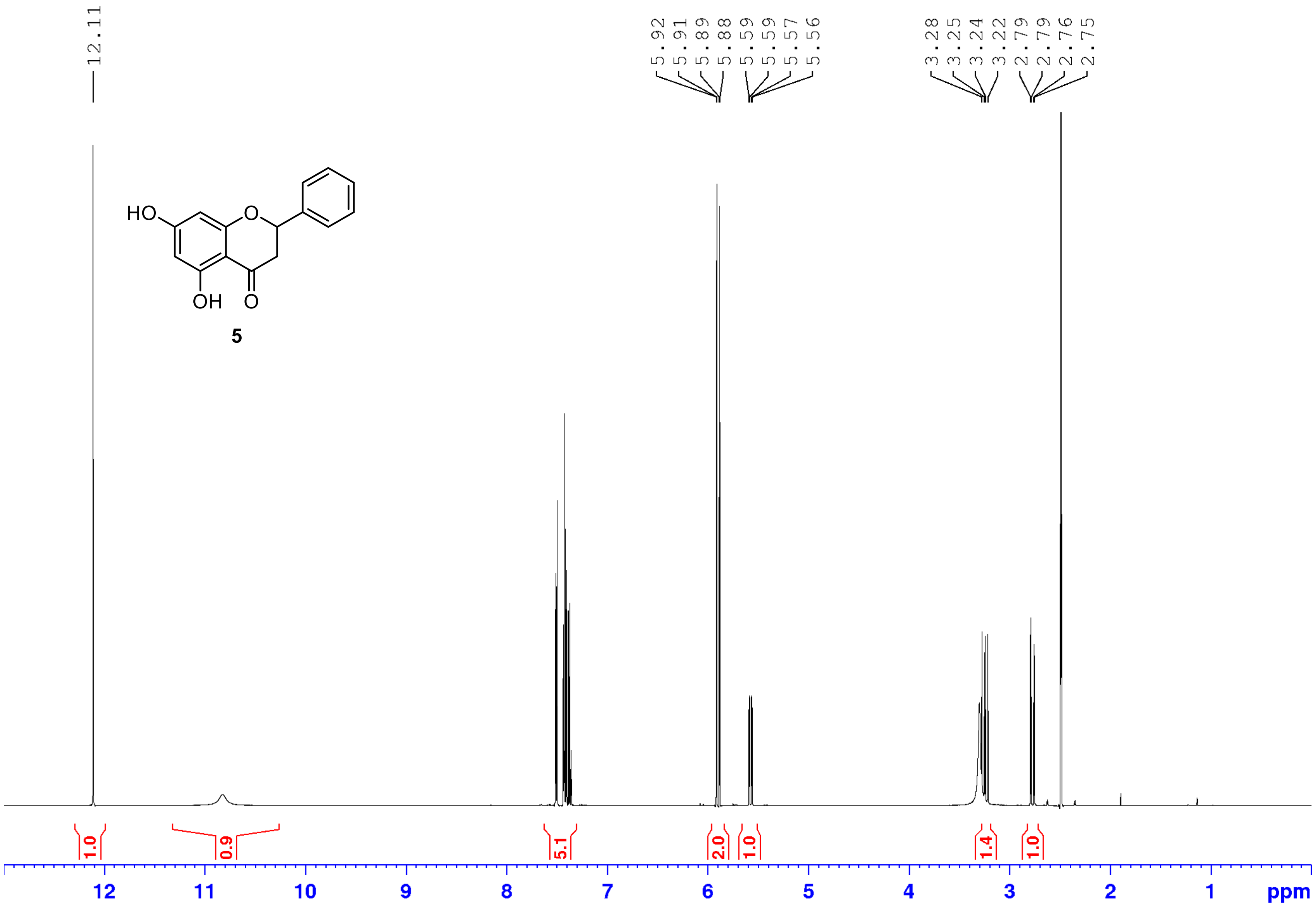

Figure S19. ^{1}H NMR spectrum of compound **5** in DMSO-d_6

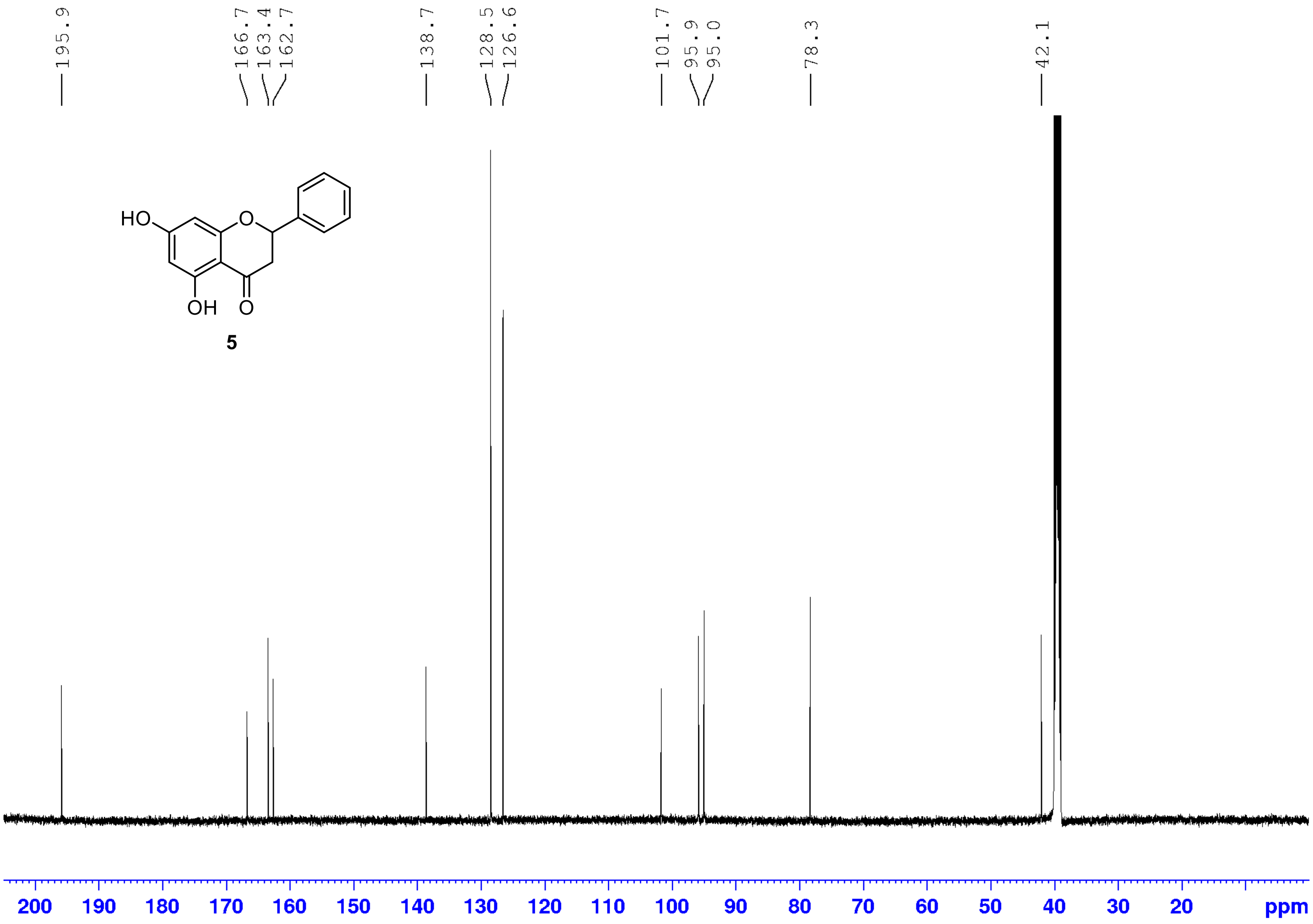

Figure S20. ^{13}C NMR spectrum of compound **5** in DMSO-d_6

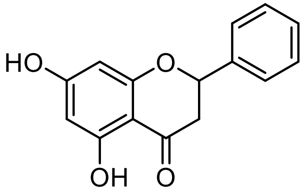

5

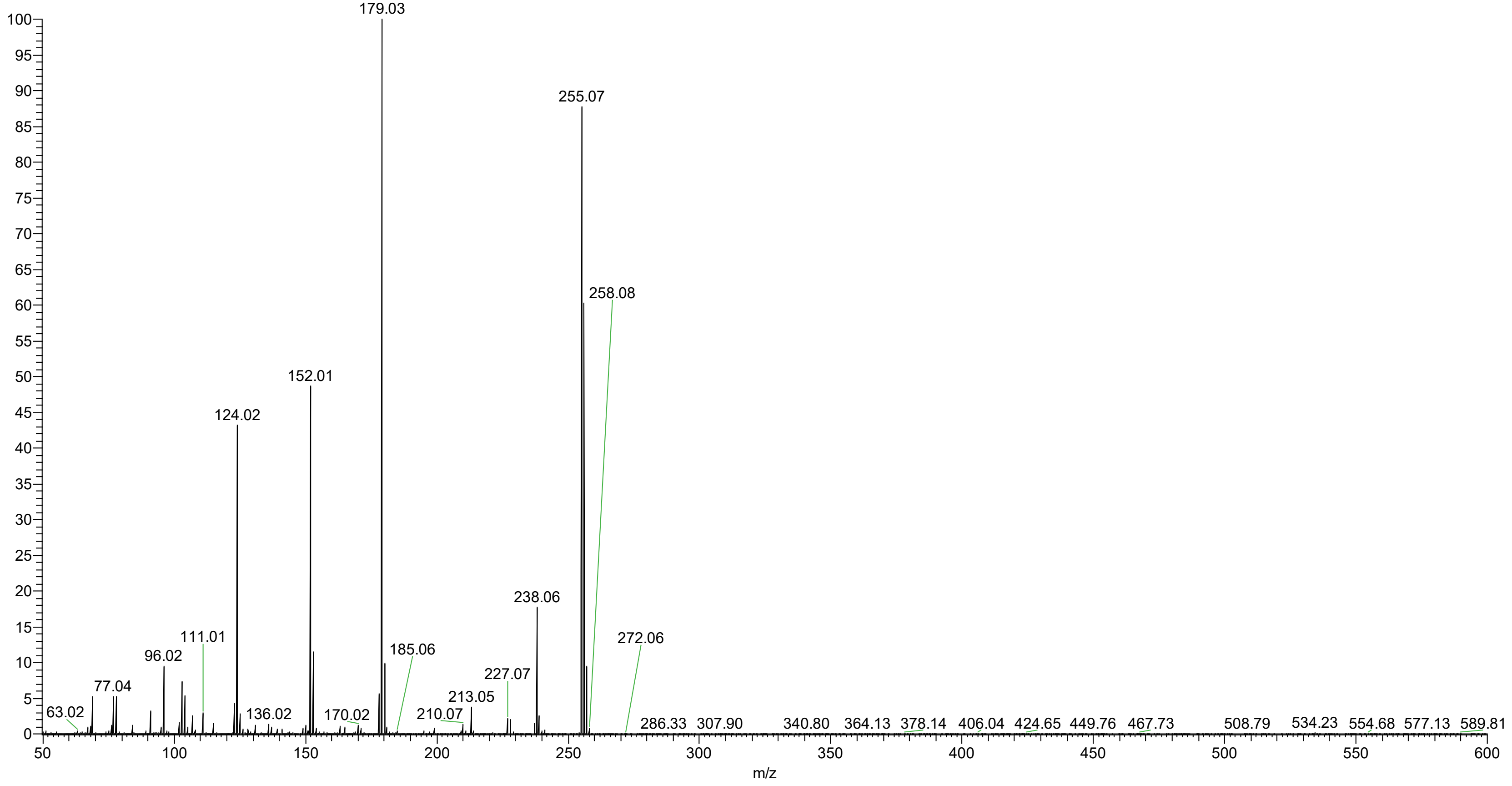

Figure S21. Mass spectrum of compound 5

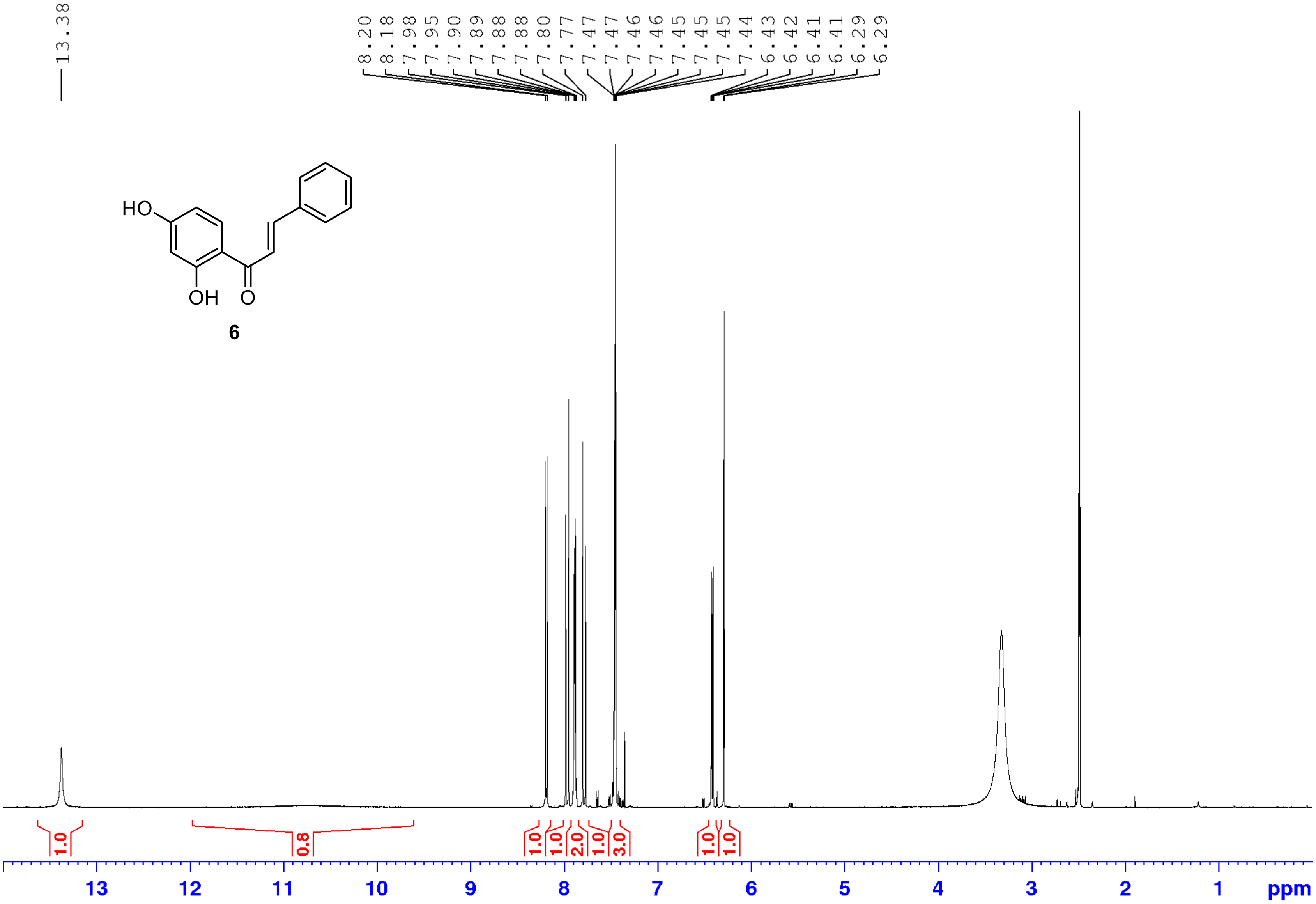

Figure S22. ^{1}H NMR spectrum of compound **6** in DMSO-d_6

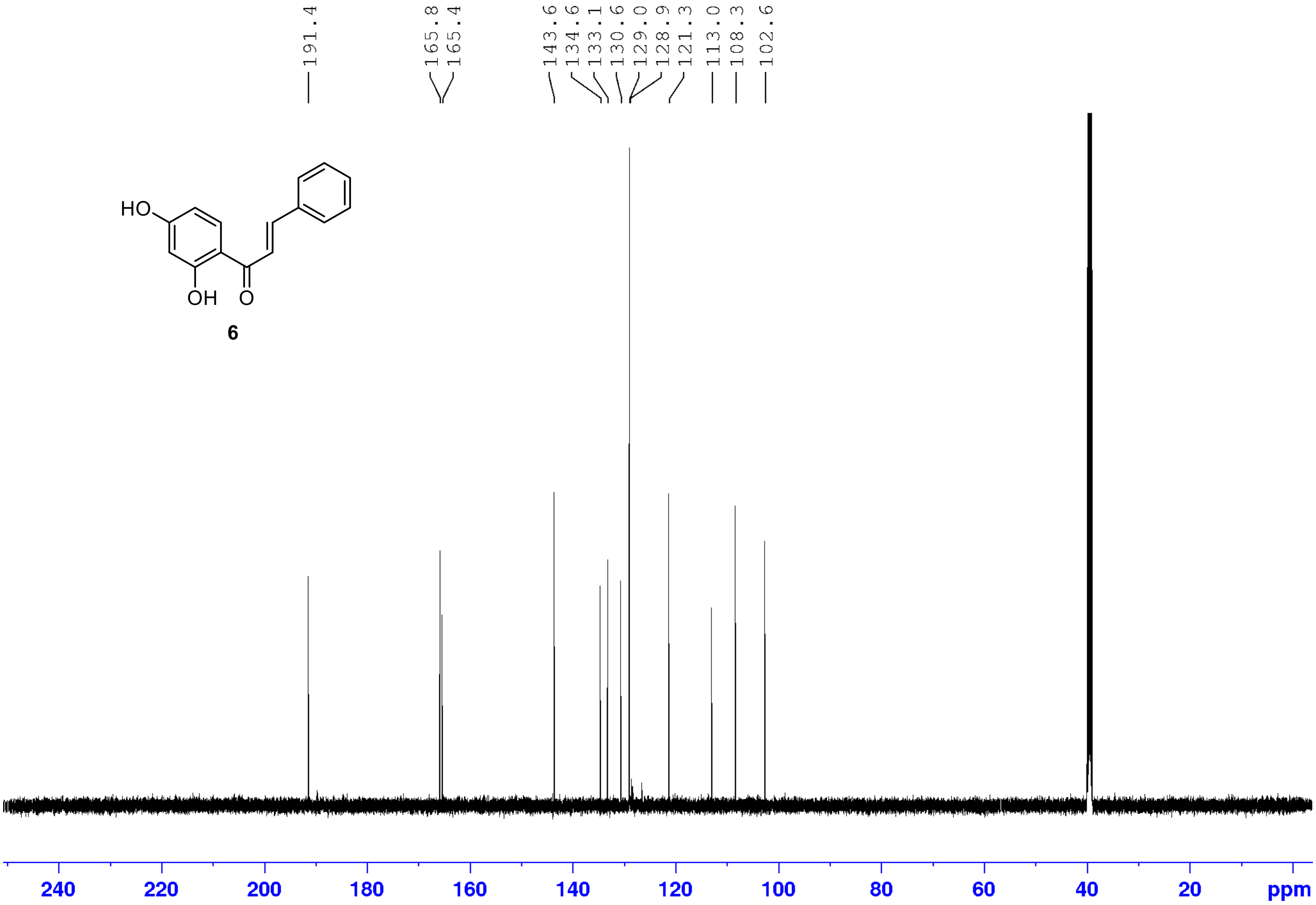

Figure S23. ^{13}C NMR spectrum of compound **6** in DMSO-d_6

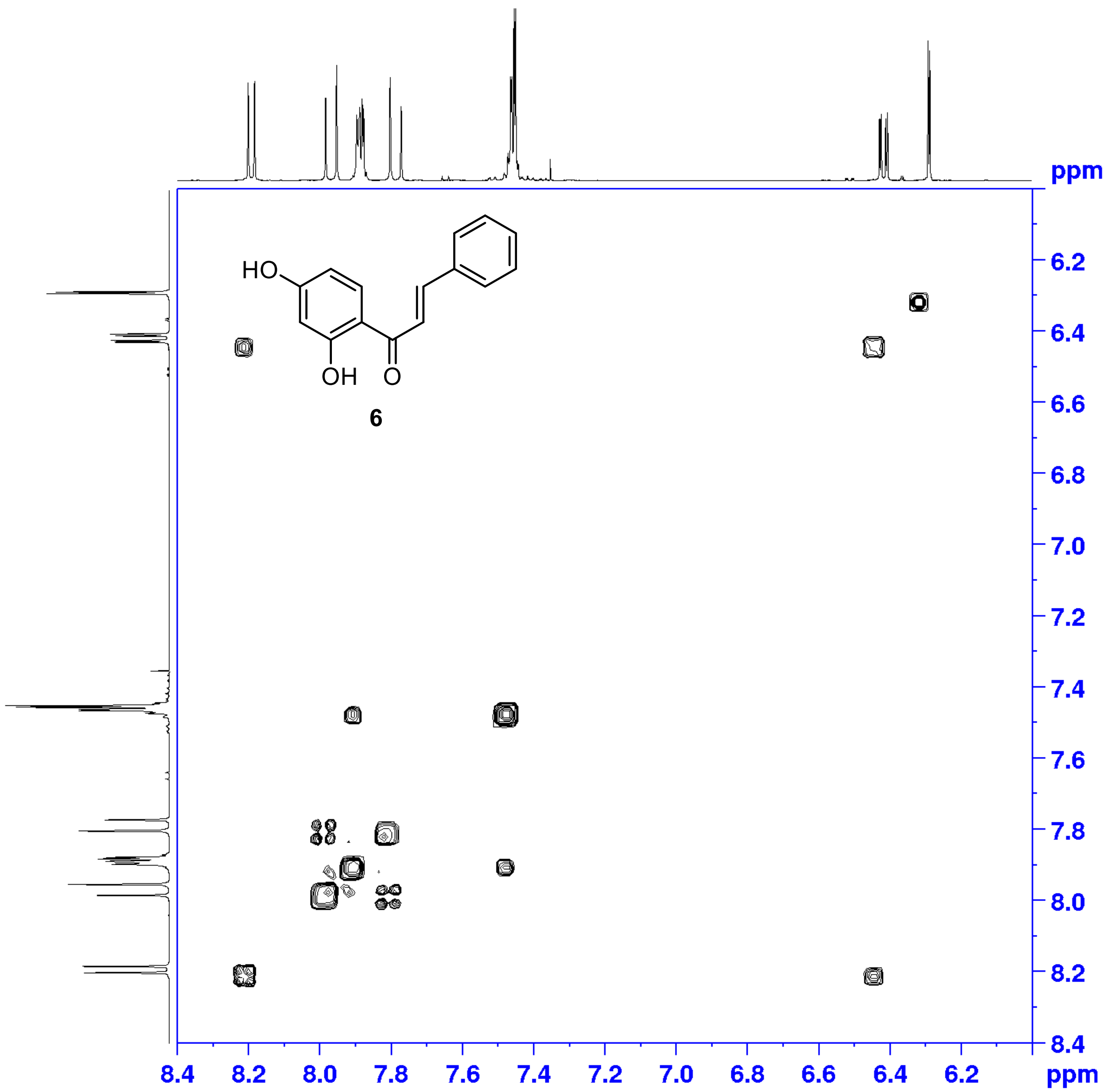

Figure S24. 2D COSY NMR spectrum of compound **6** in DMSO-d_6

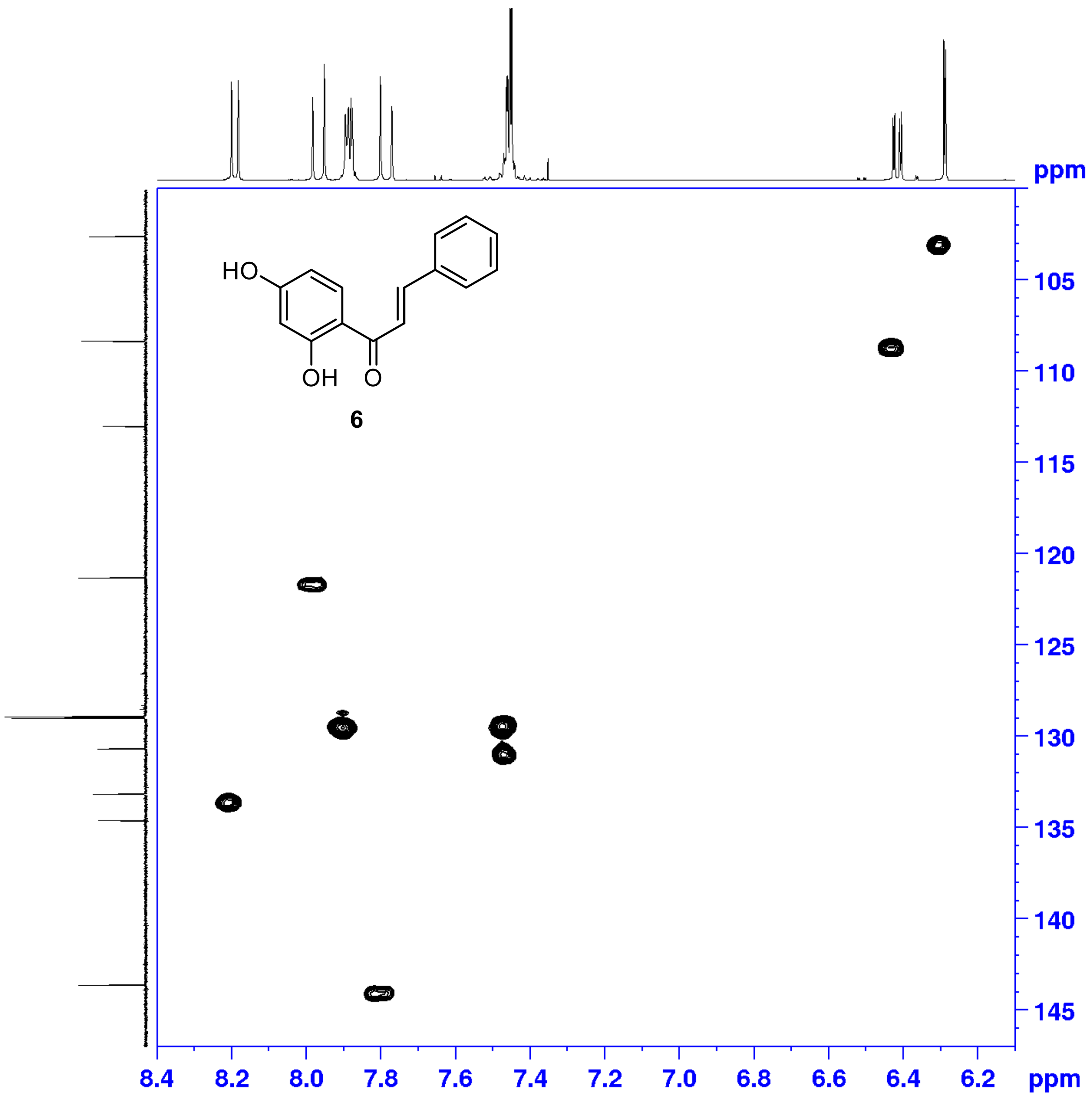

Figure S25. 2D HSQC NMR spectrum of compound **6** in DMSO-d_6

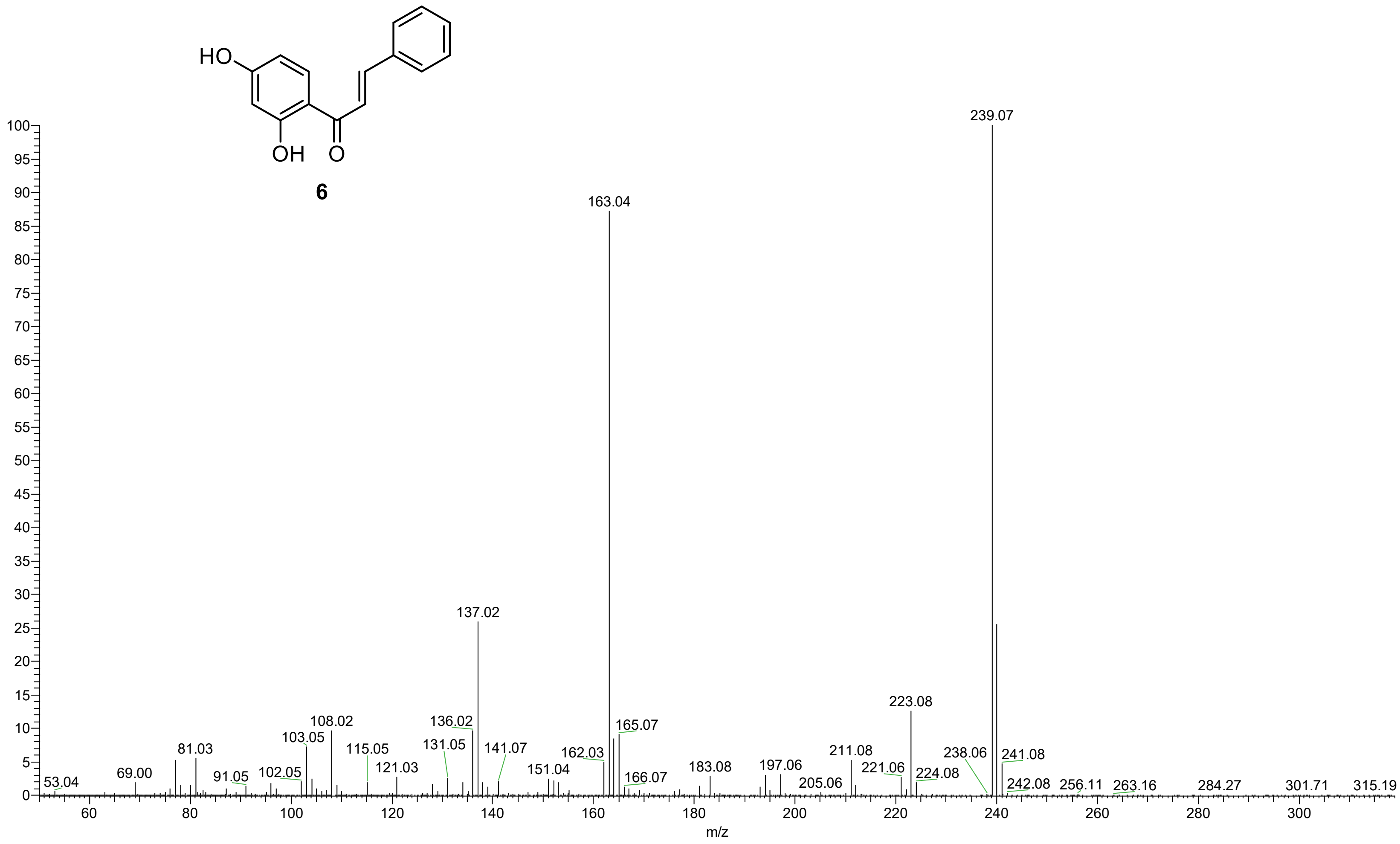

Figure S26. Mass spectrum of compound 6

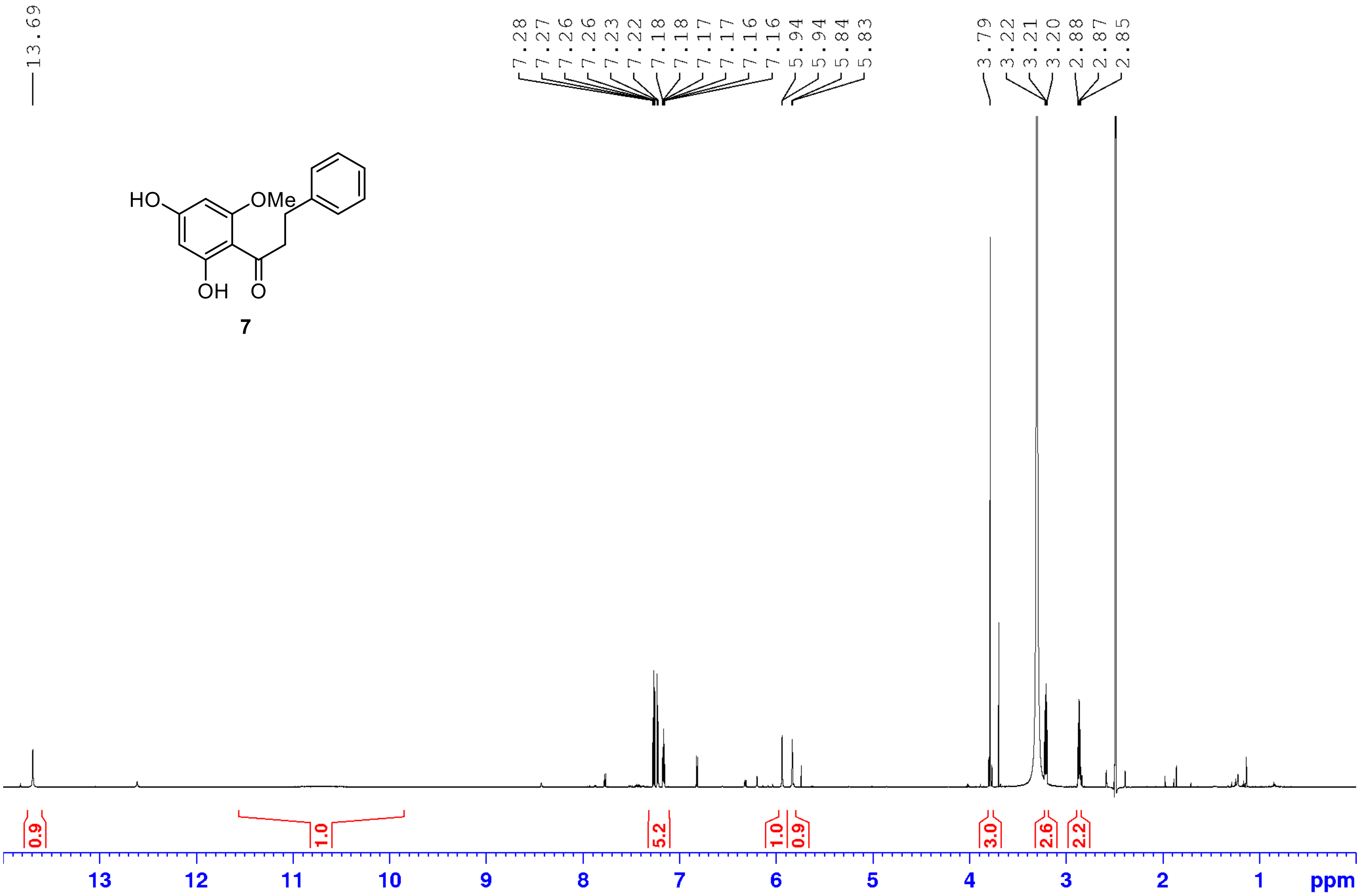

Figure S27. ^{1}H NMR spectrum of compound **7** in DMSO-d_6

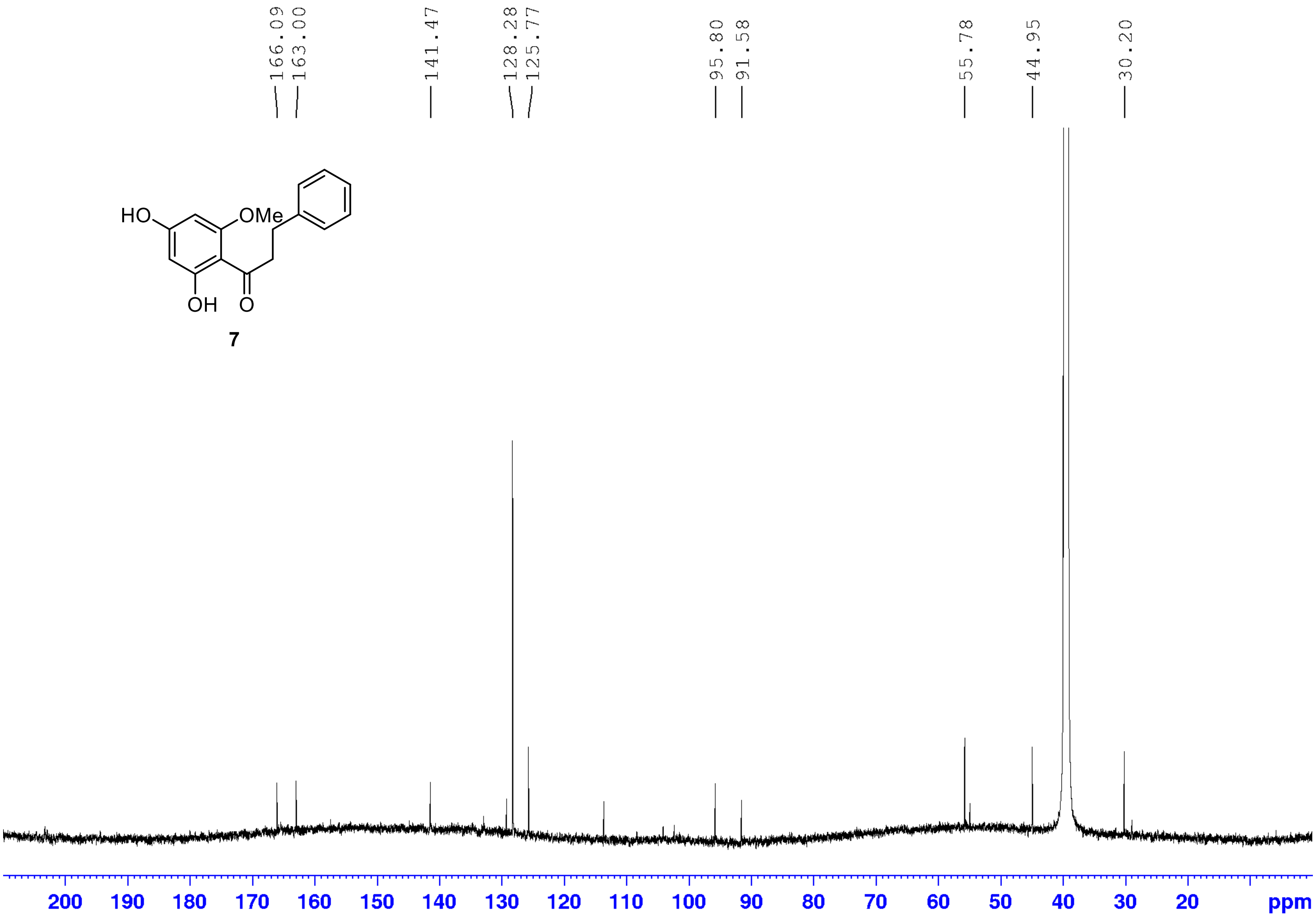

Figure S28. ^{13}C NMR spectrum of compound **7** in DMSO-d_6

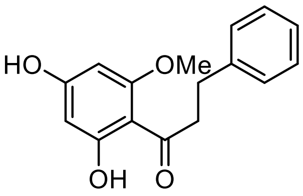

7

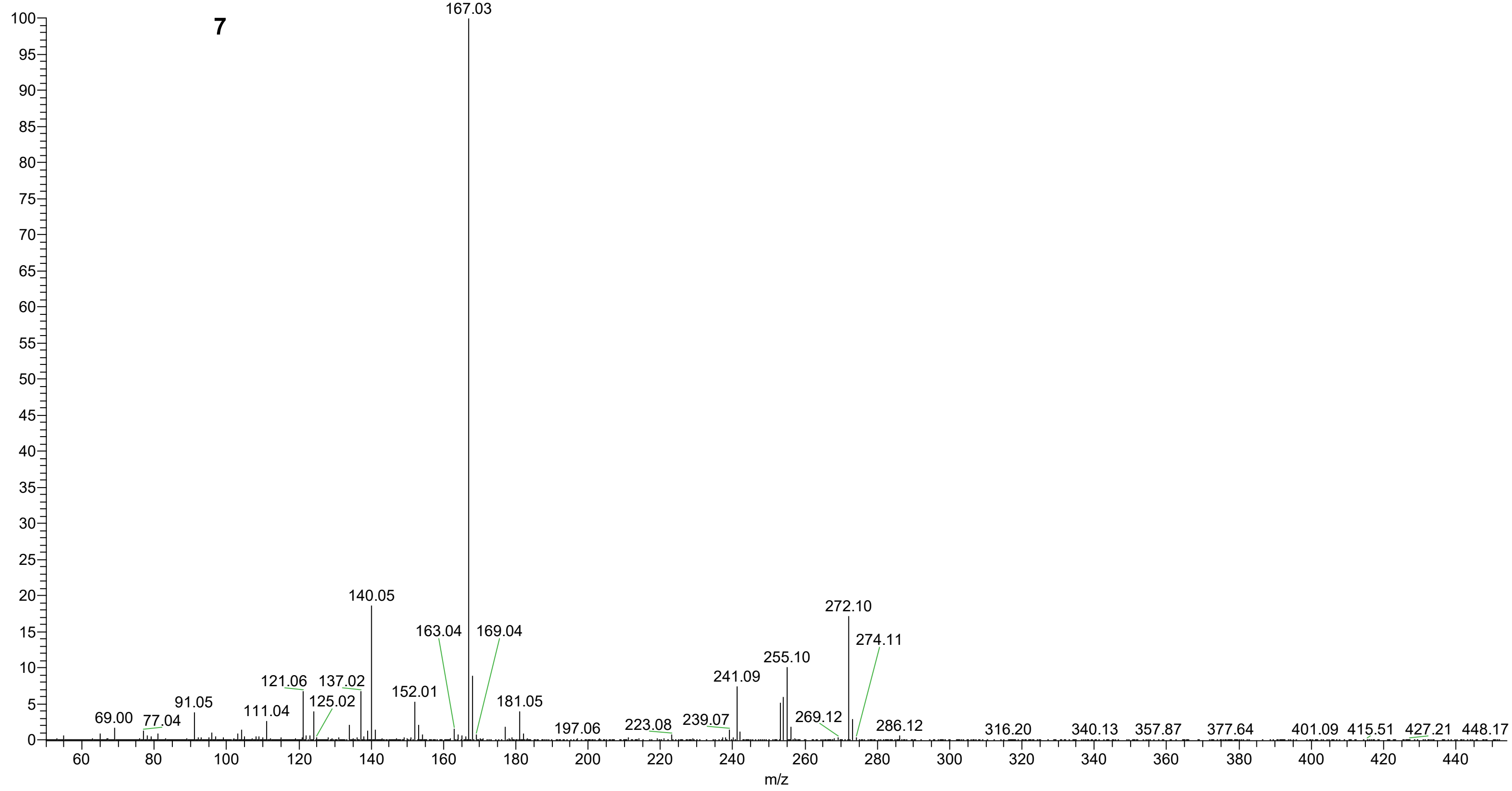

Figure S29. Mass spectrum of compound 7

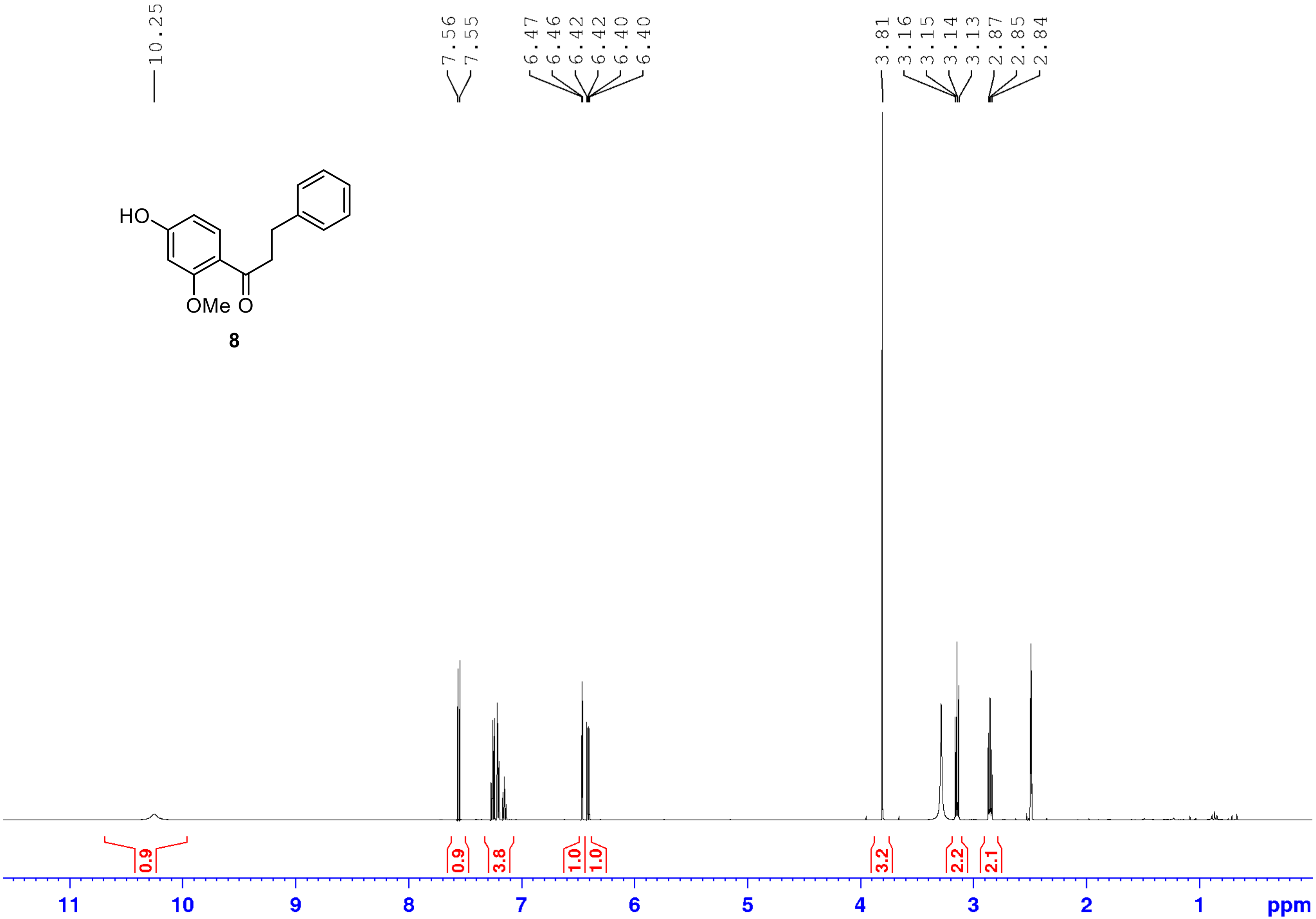

Figure S30. ^{1}H NMR spectrum of compound **8** in DMSO-d_6

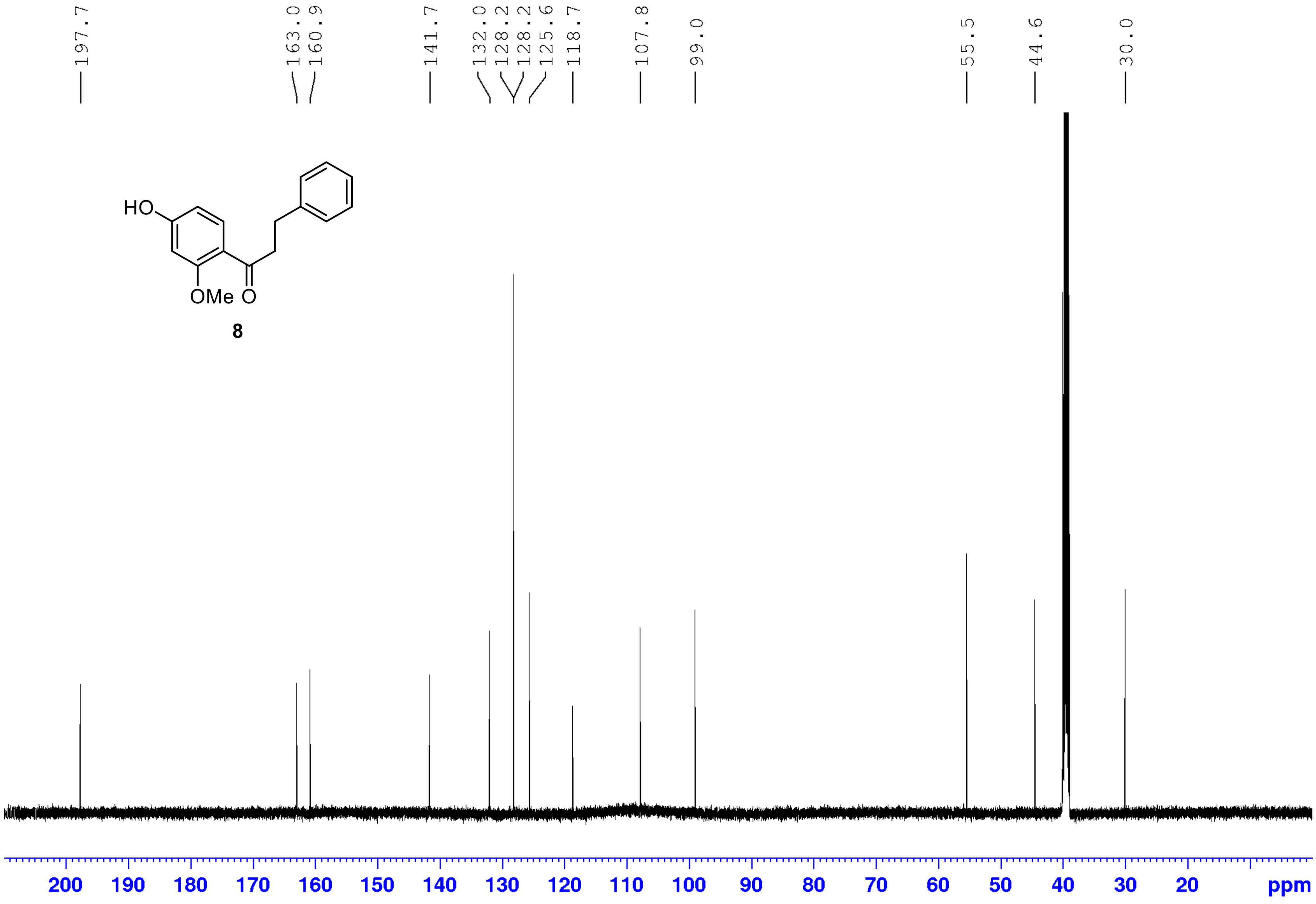

Figure S31. ^{13}C NMR spectrum of compound **8** in DMSO-d_6

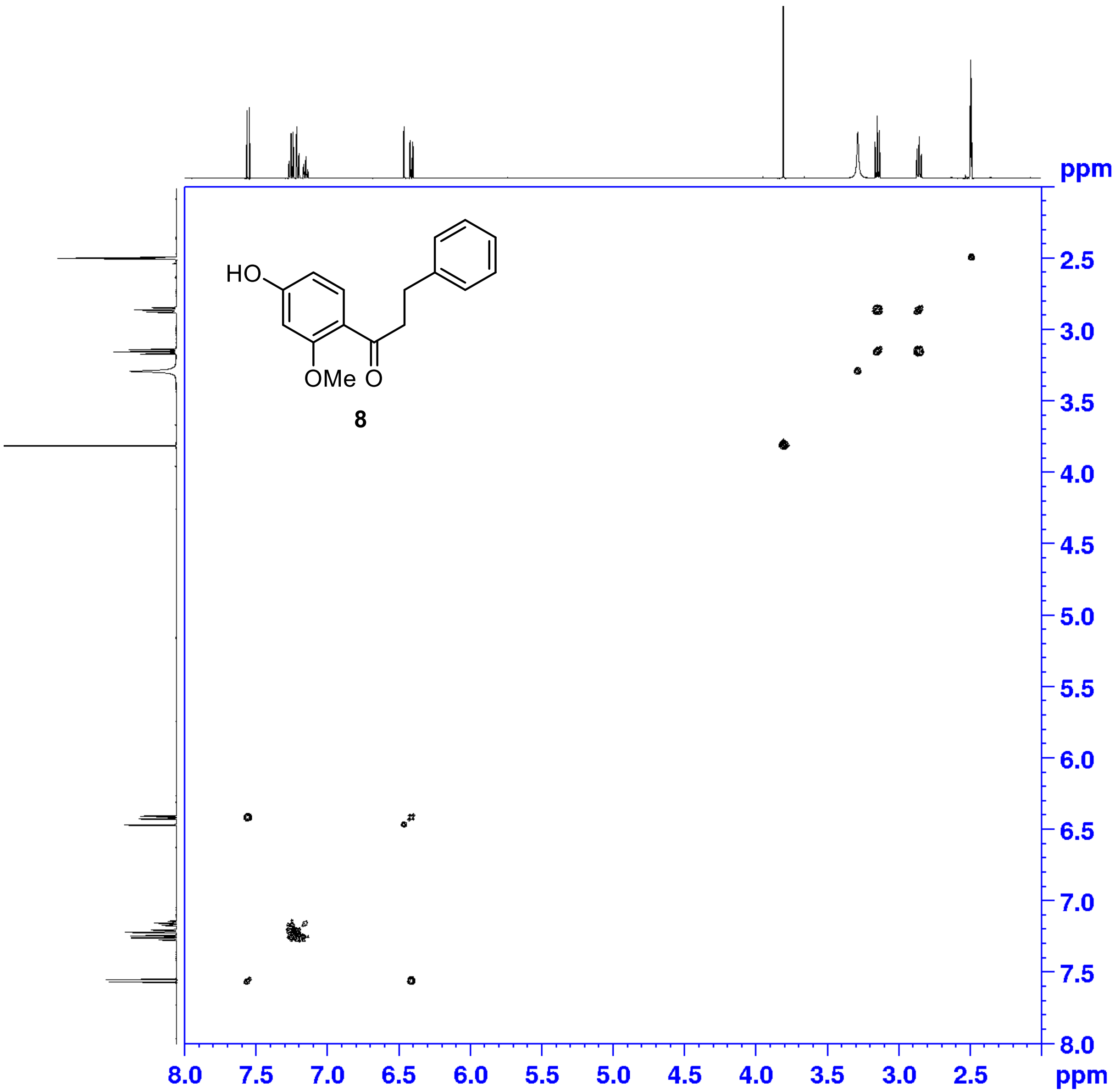

Figure S32. 2D COSY NMR spectrum of compound **8** in DMSO-d_6

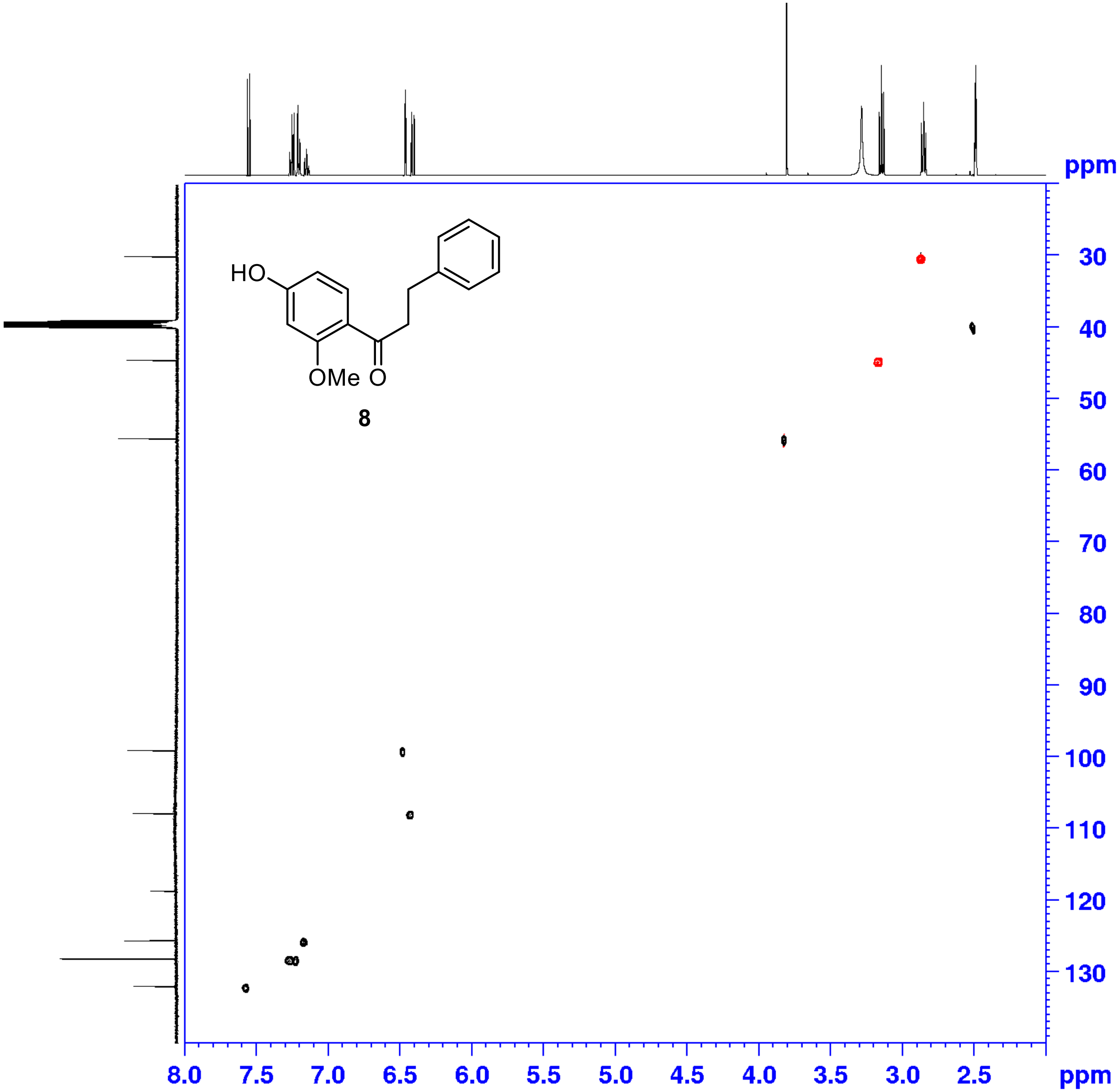

Figure S33. 2D HSQC NMR spectrum of compound **8** in DMSO-d_6

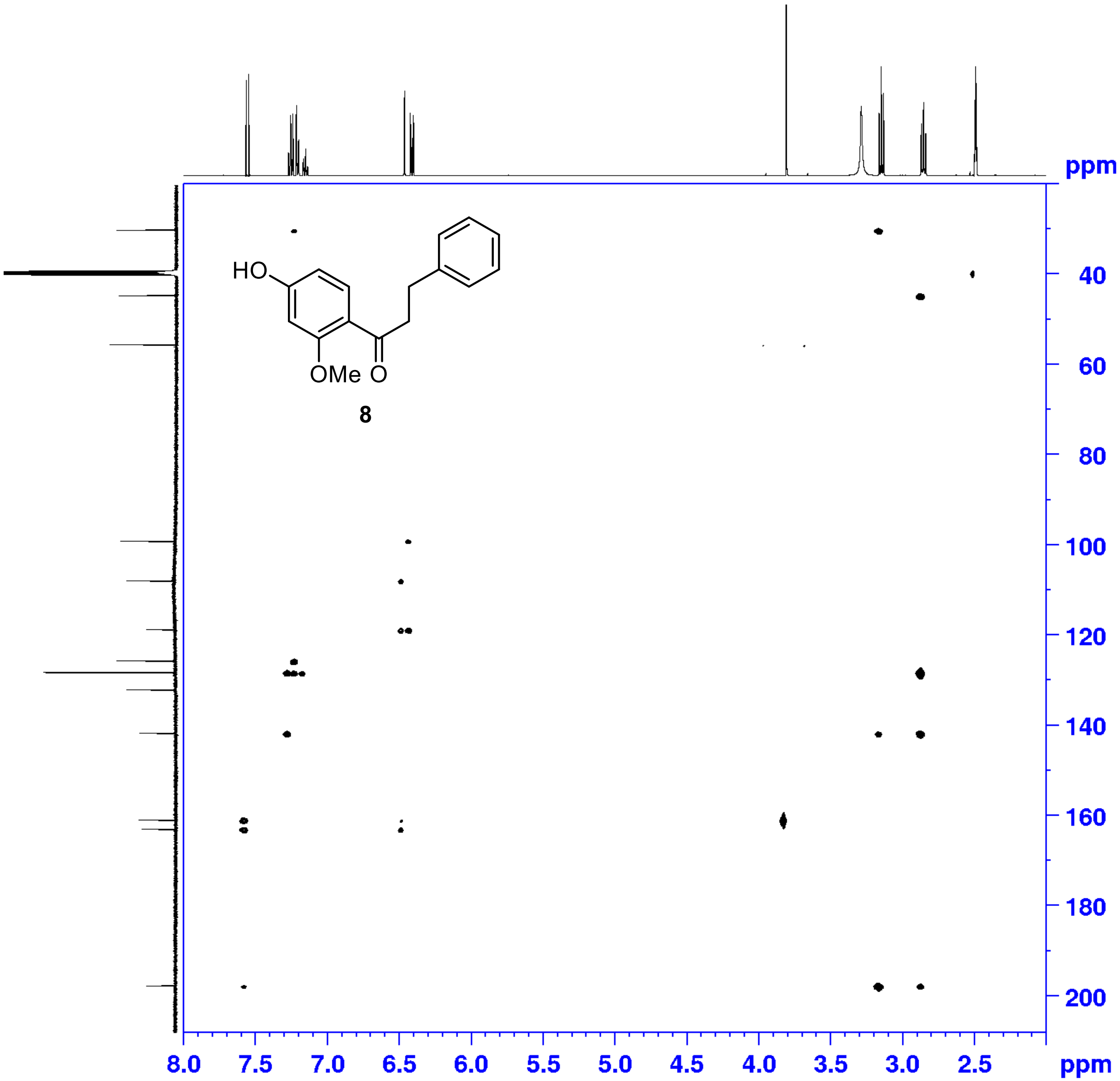

Figure S34. 2D HMBC NMR spectrum of compound **8** in DMSO-d_6

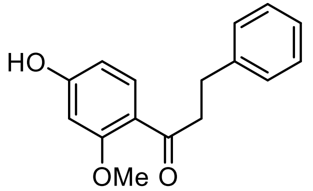

8

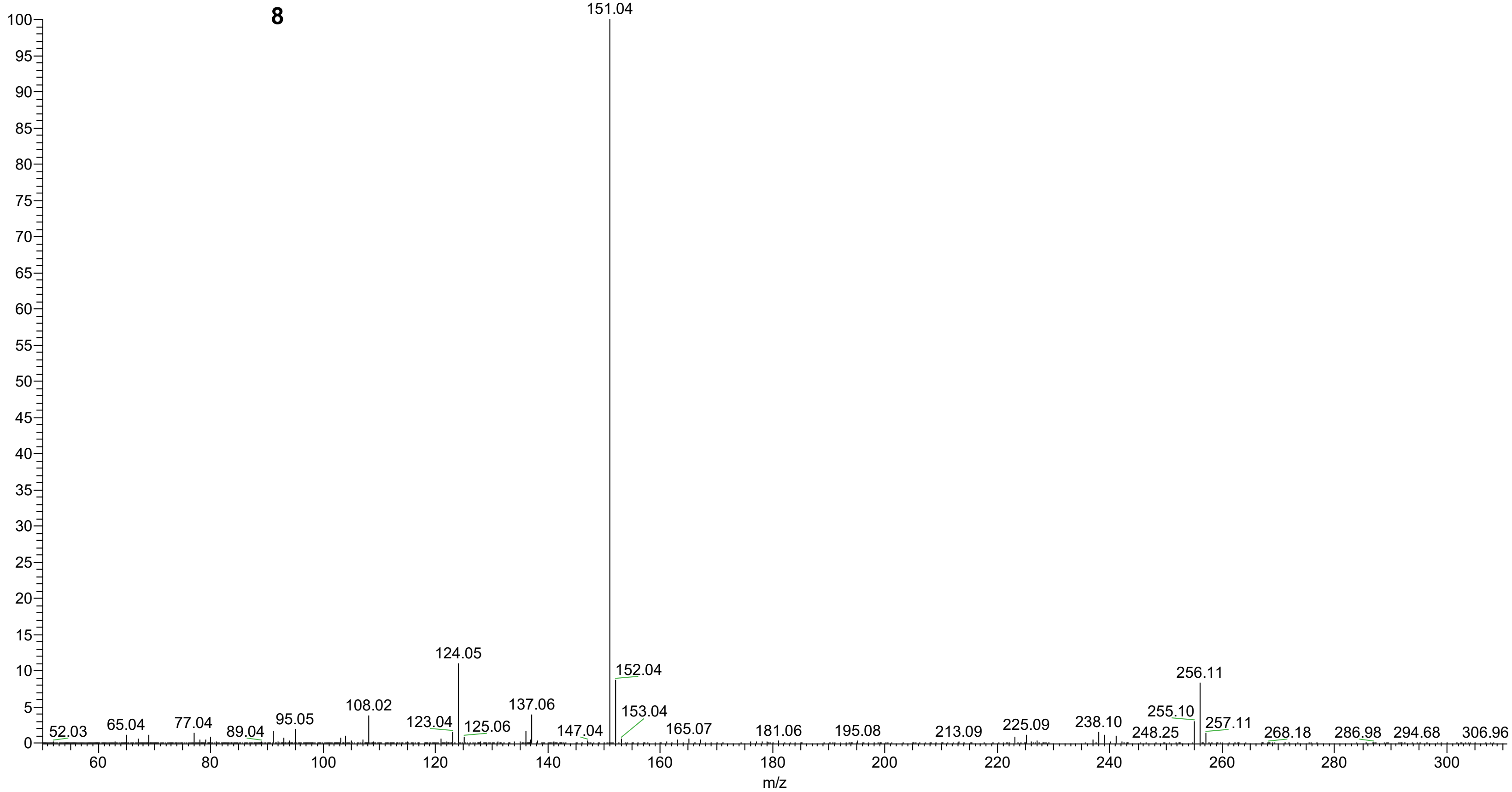

Figure S35. Mass spectrum of compound 8
